# APOE in subjective cognitive decline: a systematic review and meta-analysis

**DOI:** 10.1007/s00415-026-14077-5

**Published:** 2026-08-30

**Authors:** Paolo Alonge, Ludovico Baiamonte, Giulia Gerardi, Angelo Torrente, Eloise Lo Mauro, Nicola Veronese, Angelo Labate, Roberto Monastero

**Affiliations:** 1 Department of Biomedicine, Neuroscience and Advanced Diagnostics, University of Palermo, Via del Vespro 129, 90127 Palermo, Italy; 2 Faculty of Medicine, Unicamillus University, Via di Sant’Alessandro 8, 00131 Rome, Italy; 3 Centre for Memory Disorders, University Hospital “Paolo Giaccone”, Via Gaetano La Loggia n, 1, 90129 Palermo, Italy

**Keywords:** Cognitive impairment, Alzheimer's disease, Dementia, Epidemiology, Genetic risk factor, Apolipoprotein E ε4 allele

## Abstract

**Background:**

Alzheimer’s disease (AD) is increasingly conceptualised as a biological and clinical continuum that includes Subjective cognitive decline (SCD), mild cognitive impairment (MCI), and overt dementia. We conducted a systematic review and meta-analysis to assess the prevalence of APOE ε4 allele in individuals with SCD.

**Methods/aims:**

Main databases were searched to identify studies published up to 15 April 2026, plus citation checking. Eligible studies included participants with SCD defined according to standardized criteria, with available APOE genotype data. Application of SCD-*plus* criteria was also recorded.

**Results:**

Of 474 screened records, 49 studies were included in the quantitative synthesis. The pooled prevalence of APOE ε4 allele carriers was 28.3% (95% CI 25.1–31.8%) in SCD, 41.0% (95% CI 35.3–46.8%) in MCI, and 22.0% (95% CI 18.6–25.9%) in cognitively normal (CN) individuals. Multivariate analysis showed that the odds of being an APOE ε4 allele carrier were significantly higher in SCD compared to HC (OR = 1.28, 95% CI 1.12–1.46, *p* <0.001) and higher in MCI compared to SCD (OR = 1.48, 95% CI 1.23–1.76, *p* <0.0001). Meta-regression analyses indicated that age and education contributed to heterogeneity in SCD cohorts, while sex distribution did not. Sensitivity analyses restricted to SCD-*plus* studies confirmed the robustness of these findings.

**Conclusion:**

The prevalence of APOE ε4 allele in SCD falls between that observed in CN and MCI populations, supporting its role as an intermediate stage in the AD continuum. However, substantial heterogeneity persists, highlighting the need for more accurate stratification approaches integrating genetic and clinical markers.

**Supplementary Information:**

The online version contains supplementary material available at https://doi.org/10.1007/s00415-026-14077-5.

## Introduction

Alzheimer’s disease (AD) is the leading cause of dementia worldwide accounting for approximately 60% of all dementia cases, with a steadily increasing prevalence due to population aging. [1, 2] AD is now regarded as a continuous spectrum, ranging from preclinical stages through mild cognitive impairment (MCI) to overt dementia, and reflecting the progressive nature of its underlying pathology. [3] According to the revised criteria for diagnosis and staging of AD from the Alzheimer's Association Workgroup, clinical staging for individuals on the AD continuum begins with an asymptomatic stage (stage 1), characterised by the presence of biomarkers of amyloidosis and tauopathy, followed by a transitional phase (stage 2) in which positive biomarkers are associated with very mild cognitive changes (often perceived subjectively by the patient), with minimal impact on daily functioning and yet with cognitive performance that remains within the normal range on standardized tests. [4] This stage is clinically consistent with the syndrome of subjective cognitive decline (SCD), which is considered a potential preclinical manifestation on the AD continuum. [5] Following the onset of SCD, cognitive decline with early functional impairment—known as Mild Cognitive Impairment (MCI)—develops, which is generally considered the first clinical stage of AD.

In recent years, the preclinical stages of cognitive decline have gained growing attention in clinical research. Indeed, most intervention studies show that early interventions are more effective in slowing the progression of cognitive decline. [6, 7] However, research on SCD has long been complicated by the absence of a universally accepted set of diagnostic criteria. The definition of SCD significantly varied across studies, and other terms including “subjective memory impairment” (SMI) and “subjective cognitive complaints” (SCC), were inconsistently defined and used interchangeably. To overcome this conceptual ambiguity, Jessen et al. formally defined the construct of SCD in 2014, finally providing a unified framework. SCD was defined as a persistent decline in cognitive functioning, reported by the individual or a close informant—most commonly affecting memory, but potentially involving other cognitive domains—in individuals with normal performances on standardised neuropsychological tests and without abnormalities on neurological examination [5].

Alongside a unified terminology, Jessen et al. proposed a set of more specific features—collectively referred to as SCD-*plus* criteria—that increase the likelihood of underlying preclinical AD in individuals with SCD. These include an age at onset of 60 years or older, symptom onset within the preceding five years, self-perceived cognitive decline compared to age-matched individuals, and subjective concern associated with the reported decline. Despite the adoption of this refined definition, biomarker findings and progression patterns in SCD populations remain heterogeneous. This variability likely reflects residual differences in the application of inclusion criteria, methodological heterogeneity across studies, and the limited precision of criteria to discriminate between progressive and non-progressive causes of cognitive decline [5, 8, 9].

In this context, genetic risk factors can serve as a key tool for stratification with the aim of reducing biological heterogeneity within SCD cohorts. Among these, the APOE ε4 allele, in either the heterozygous (ε4+/−) or homozygous (ε4+/ +) forms, is the well-established genetic risk factor for sporadic AD, implicated in amyloid metabolism, tau pathology, neuroinflammation, and blood–brain barrier dysfunction. [10–12] Although the role of APOE ε4 is well established in AD and MCI, its relevance in SCD remains debated. Previous studies and reviews have yielded inconsistent findings, with some reporting no difference in APOE ε4 prevalence between individuals with SCD and healthy controls. Although this may seem unexpected, given the recognised role of SCD as a potential preclinical stage of AD and the consistently high frequency of APOE ε4 observed in MCI and AD dementia, these discrepancies may reflect heterogeneity in how SCD has been defined in studies conducted prior to the current diagnostic criteria for SCD [13].

Since the introduction of the SCD framework diagnostic criteria, several studies have investigated the role of genetic factors, particularly APOE, in the incidence and risk of disease progression in SCD. To summarise these findings, a systematic review and meta-analysis were conducted to comprehensively assess the prevalence of APOE4 in subjects with SCD and its role in predicting the cognitive status (SCD vs MCI and SCD vs normal cognition).

## Methods

We defined our research question according to the PECO (population, exposure, comparator, outcome) framework and designed our research protocol accordingly [14].

- *Population* individuals with SCD, according to the inclusion criteria mentioned above. [5] When included studies also reported data on CN or MCI participants, these groups were additionally considered as comparison groups. Studies focusing exclusively on CN or MCI without an SCD group were not eligible for inclusion.
- *Exposure* Carriership of at least one APOE ε4 allele.
- *Comparator* Non-carriers of the APOE ε4 allele within each diagnostic group. For pairwise analyses, SCD served as the reference group against which CN and MCI were compared.
- *Outcome* Proportion of APOE ε4 carriers in each diagnostic group (SCD, MCI, CN), and odds ratios for the presence of the APOE ε4 gene when comparing CN vs SCD and MCI vs SCD.

A systematic review of the literature was conducted and the data collected were pooled in a meta-analysis. The results are reported in accordance with the Preferred Reporting Items for Systematic reviews and Meta-Analyses (PRISMA) 2020 guidelines [15].

### Eligibility criteria

The following inclusion criteria were adopted: (1) adopting a cross-sectional or longitudinal design (both retrospective and prospective). From longitudinal studies, only baseline data from SCD, CN, or MCI populations were included, without considering longitudinal progression; (2) reporting data on patients with SCD, as defined below; (3) providing data on the APOE genotype status of the participants.

SCD definition was considered adequate for inclusion when it fulfilled the following criteria: (1) cognitive complaint in one or more domains reported by the patient and/or an informant, or identified through a validated questionnaire, such as the Memory Complaint Questionnaire (MAC-Q) or the Subjective Cognitive Decline-Questionnaire (SCD-Q) [16, 17]; (2) exclusion of overt cognitive decline (i.e. MCI or dementia) using formal neuropsychological testing; (3) exclusion of other psychiatric disorders or neurological and/or systemic conditions that could potentially interfere with cognitive performance. Studies using older classifications, such as Subjective Memory Complaints (SMC) or Subjective Memory Impairment (SMI), were included only if their definition fulfilled the up mentioned criteria. Studies reporting mixed populations (e.g. MCI and SCD) were included only if it was possible to extract data specific to subjects with SCD.

Furthermore, we excluded: (1) book chapters, reviews, letters, case reports or conference abstracts; (2) studies not providing enough data to calculate the prevalence of APOE4 status in SCD subjects; (3) studies where SCD was not clearly defined as stated above; (4) studies with non-English full text.

To manage the unit-of-analysis problem, when multiple studies from the same database were found, we included only the largest one.

### Search strategy

A systematic literature search was conducted on MEDLINE (via PubMed), Web of Science and Scopus databases from inception to April 15, 2026, using search strategies tailored to each database (see Supplementary Material, Table S1). The reference lists of eligible articles and relevant reviews (backward/forward citation checking) were also reviewed. The research output was checked for duplicates, subsequently two authors (P.A. and G.G.) independently screened the studies against the inclusion criteria, providing an inclusion or exclusion judgement. In case of discrepancy, a third senior author (R.M.) made the final decision regarding the inclusion or exclusion of the study. The same process was repeated for the abstracts and subsequently for the full text articles.

### Data extraction

Data were extracted from the selected studies by two authors (P.A. and G.G.), using a predefined database electronic sheet. The following variables were extracted: study design, publication date, number of participants with SCD, MCI and/or CN participants, age, sex, educational level, prevalence of the APOE ε4 genotype, sample type (population-based, hospital-based), and diagnostic criteria used for SCD. Data were reported as mean ± standard deviation (SD) or median ± interquartile range (IQR). Where available, range for age of participants was included.

### Risk of bias assessment

The risk of bias in the included studies was assessed using the critical appraisal checklists provided by the Joanna Briggs Institute (JBI) for prevalence studies. As the proportion of APOE ε4 carriers was a secondary outcome in most of the included studies, each item on the JBI was assessed with specific reference to the quality of the reported APOE ε4 data, rather than to the overall study methodology [18].

Publication bias was assessed using Egger's test for funnel plot asymmetry. [19] An intercept <0.10 was considered indicative of funnel plot asymmetry. In addition, Duval and Tweedie's “trim-and-fill” method was applied to adjust pooled estimates, under the assumption of funnel plot asymmetry.

Furthermore, to account for the risk of outlier studies, which could disproportionately influence the overall pooled result, we performed a leave-one-out analysis to compute the following influence diagnostics for each study: the difference in fits (DFFITS); Cook's distance; hat values; and the difference in regression coefficients (DFBETAS). Each study was sequentially removed from the meta-analysis, and the model was refitted on the remaining k−1 studies. A study was considered significant when at least one of the following criteria was met: (i) the absolute DFFITS value exceeded $3\sqrt{\frac{\mathrm{p}}{\mathrm{k}-\mathrm{p}}}$, where p is the number of model coefficients and k the number of studies; (ii) the lower tail area of a chi-square distribution with p degrees of freedom, cut off by Cook's distance, exceeded 50%; (iii) the hat value exceeded$3\frac{p}{k}$; (iv) any absolute DFBETAS value exceeded 1. These diagnostics were applied separately to each of the five meta-analytic models (proportion of APOE ε4 carriers in SCD, CN, and MCI; odds ratios for CN vs SCD and MCI vs SCD).

### Meta-analysis

A meta-analysis was conducted to obtain a pooled estimate of the proportion of APOE ε4 carriers in subjects with SCD. A random-effects model was used, employing the restricted maximum likelihood method; to ensure compliance with the normality assumptions, the response variable was logit-transformed before being entered in the model. Two-sided p-values of 0.05 or less were considered statistically significant, and results were presented using a forest plot. Statistical heterogeneity among the selected studies was assessed using the Cochran's Q statistic (*p* values <0.10 were considered indicative of statistically significant heterogeneity) and the *I*^2^ statistic. RStudio version 2023.12.1 with R version 4.3.2 was used for the analyses; finally, the Metafor package was used [20–22].

To identify possible sources of heterogeneity, we performed a meta-regression to assess the role of the proportion of female participants, the mean age of participants, and the years of education of participants in the included studies. To this scope, these covariates were included as moderator variables in the random-effects model, thereby obtaining a mixed-effects model. Each group (SCD, MCI and healthy controls) was analysed separately. The proportion of female participants was categorised as above or below 50%.

In addition, the odds of being an APOE ε4 carrier were compared pairwise across the three groups (CN vs SCD and MCI vs SCD). Specifically, in each included study, the odds ratios (ORs) with 95% confidence intervals (95% CI) for these comparisons were calculated (only studies that allowed for at least one of these comparisons were included); subsequently, they were pooled using a multivariate model via the Mixmeta package. [23] This model allowed to jointly synthesise multiple, correlated effect sizes (in this case two ORs with the same denominator, namely people with SCD), accounting for within-studies correlations, since the variance–covariance matrix of ORs was included as a random effect and the matrix of ORs as the outcome.

## Results

**Fig. 1:**
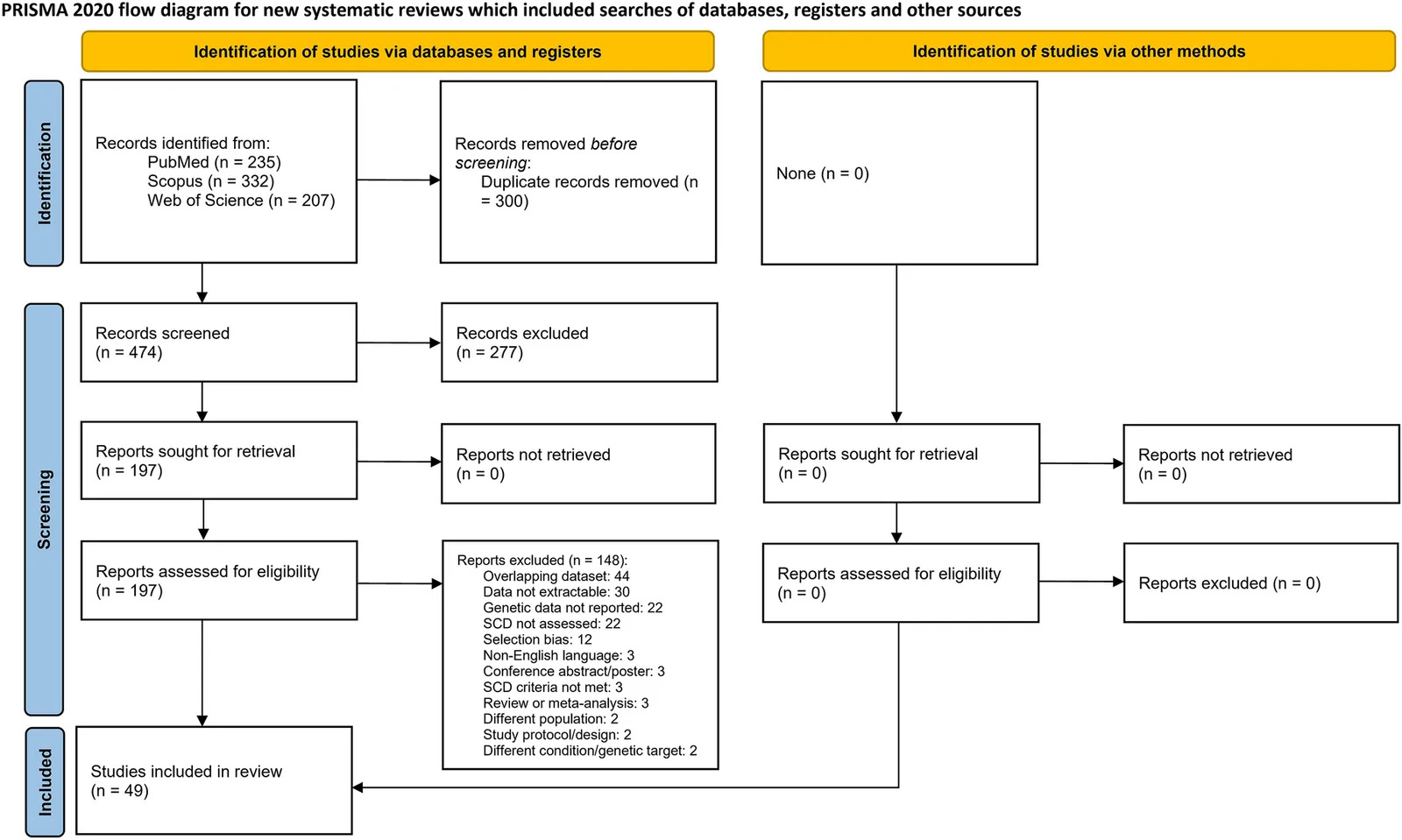
PRISMA flow diagram in the study selection process. Source: Page MJ, et al. BMJ 2021;372:n71. https://doi.org/10.1136/bmj.n71. This work is licensed under CC BY 4.0. To view a copy of this license, visit

Initially, 774 articles were identified, of which 474 remained after duplicate removal. After an initial screening based on titles, 197 were selected based on abstracts and assessed for eligibility. Ultimately, forty-nine studies met the inclusion criteria and were included in the present review. For details on the study selection process, see Fig. 1. A full list of excluded abstracts with the corresponding reason for exclusion is available in Supplementary Materials.

### Characteristics of the included studies

All 49 studies were included in the quantitative synthesis of data on the prevalence of the APOE ε4 allele. Homozygous/heterozygous APOE ε4 status was not analysed as it was not reported consistently across studies. Of the included studies, 26 were cross-sectional, whilst 23 were prospective. Twenty-six were conducted in European countries, 7 in North America, 13 in Asia (with China being the most involved country), 2 in Australia and one in the Middle East. The publication years ranged from 2005 to 2026. For more details on the included articles, see Supplementary Material, Table S2, S3.

Of the cross-sectional studies included, only 3 directly evaluated the role of the APOE ε4 allele. [24–26] Specifically, Hao et al. reported a higher prevalence of APOE ε4 carriers in SCD compared to CN subjects; furthermore, APOE status was associated with the severity of self-reported cognitive complaints. [24] Hestad et al. found lower cognitive performance in APOE ε4 carriers compared to non-carriers with SCD. [25] In contrast, De Rojas et al. reported no significant association between APOE genotype and amyloid burden in individuals with SCD [26].

Regarding the remaining studies, 10 examined the reliability and distribution of biomarkers across the AD spectrum, [27–36] whilst 6 assessed the discriminative utility of structural and functional neuroimaging across the AD continuum. [37–42] Three studies examined neuropsychological performance between individuals with SCD and CN controls, [43–45], whilst 2 focused on the incidence of SCD in selected populations. [46, 47] Finally, 2 studies explored the contribution of lifestyle-related risk factors. [48, 49]. Detailed information on the main assessments and principal findings of each cross-sectional study is reported in Supplementary Material (Table S2).

Among the longitudinal studies, 3 specifically examined the role of APOE ε4 in individuals with SCD. [50–52] Four studies developed predictive models to estimate the risk of progression from SCD to MCI, [53–56] while 2 others analysed the impact of disclosing APOE status. [57, 58]. Three studies were designed as observational cohort ones. [59–61] Six studies explored risk factors and biomarkers associated with progression in SCD. [62–67] Finally, one study compared different recruitment strategies [68].

Regarding the effect of APOE on progression rates in SCD patients, the 3 studies that directly addressed this issue consistently reported an increased risk of conversion to objective cognitive decline in APOE ε4 carriers. [50–52] Similarly, observational cohort studies and studies evaluating biomarkers associated with progression have shown that APOE ε4 is associated with a higher risk of progression and greater changes in cerebrospinal fluid (CSF) and plasma biomarkers, [55, 61, 62, 64, 66] with the exceptions of Zhen Liu et al. and Sierra-Rio et al., who reported no association between APOE status and the distribution of biomarkers in CSF. [59, 65] Furthermore, studies specifically evaluating the risk of progression in SCD cohorts have not consistently reported an independent effect of APOE genotype. [53, 54, 63] Key assessments and main findings of longitudinal studies are reported in Supplementary Material (Table S3).

### Pooled proportion of APOE ε4 carriers

**Fig. 2:**
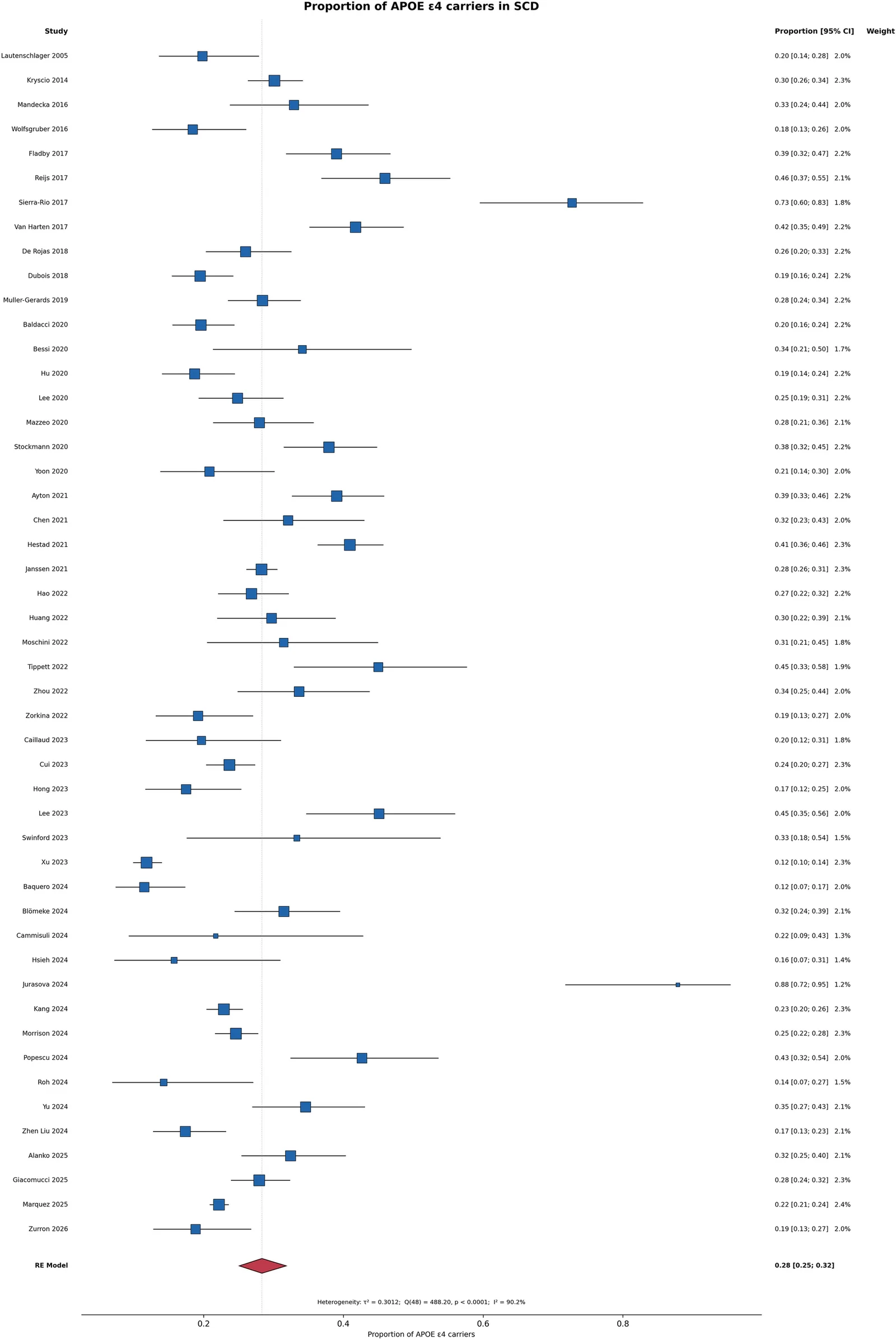
Forest plot of the pooled proportion of APOE ε4 carriers among individuals with subjective cognitive decline (*SCD*). Each square represents the study-specific proportion, with the size proportional to the study weight. Horizontal lines indicate 95% confidence intervals. The red diamond represents the pooled estimate from the random-effects model (*REML*). Studies are ordered by year of publication and alphabetically within each year

Among individuals with SCD, the estimated pooled prevalence of APOE ε4 allele was 28.3% (95% CI 25.1–31.8%). Significant statistical heterogeneity was observed (*I*^2^ = 90.2%). The results of the meta-analysis are presented in the forest plot in Fig. 2. In the meta-regression analysis, the mean age of participants showed a significant negative association with the proportion of APOE ε4 allele carriers (*β* = − 0.04, *p* = 0.014), accounting for 14.8% of the between-study variance (*R*^2^=14.8%) and reducing residual heterogeneity from *I*^2^ = 94.2% to *I*^2^_res = 93.3%. Education of participants showed a positive association (*β* = 0.09, *p* = 0.020; *R*^2^ = 11.0%; *I*^2^_res = 85.8%, from a baseline *I*^2^ = 87.1%). In contrast, the proportion of female participants did not have a significant effect on the proportion of APOE ε4 allele carriers (*β* = −0.33, *p* = 0.142; *R*^2^ = 0.1%; *I*^2^_res = 94.2%). See Supplementary Material for further details (Table S4a).

Among individuals with MCI, the pooled prevalence of APOE ε4 allele carrier status was 41.0% (95% CI 35.3–46.8%). No significant impact of the examined covariates on this proportion was observed: proportion of female participants (*β* = −0.44, *p* = 0.053; *R*^2^ = 17.2%; *I*^2^_res = 87.8%), mean age (*β* = −0.001, *p* = 0.945; *R*^2^ = 0.0%; *I*^2^_res = 90.1%), years of education (*β* = 0.08, *p* = 0.098; *R*^2^ = 14.8%; *I*^2^_res = 85.4%). See Supplementary Material (Table S4bc, Figure S1).

In CN subjects, the proportion of APOE allele ε4 carriers estimated from included studies was 22.0% (95% CI 18.6–25.9%). Both mean age (*β* = 0.05, *p* = 0.007; *R*^2^ = 50.6%; *I*^2^_res = 62.6%, down from *I*^2^ = 77.2%) and mean years of education (*β* = 0.12, *p*=0.002; *R*^2^ = 91.9%; *I*^2^_res = 6.0%, down from *I*^2^ = 44.4%) showed a positive correlation with the proportion; in contrast, the proportion of female participants had no significant effect (*β* = 0.47, *p* = 0.067; *R*^2^ = 19.9%; *I*^2^_res = 82.4%). See Table S4b in Supplementary material and Figure S2.

### Multivariate model

**Fig. 3:**
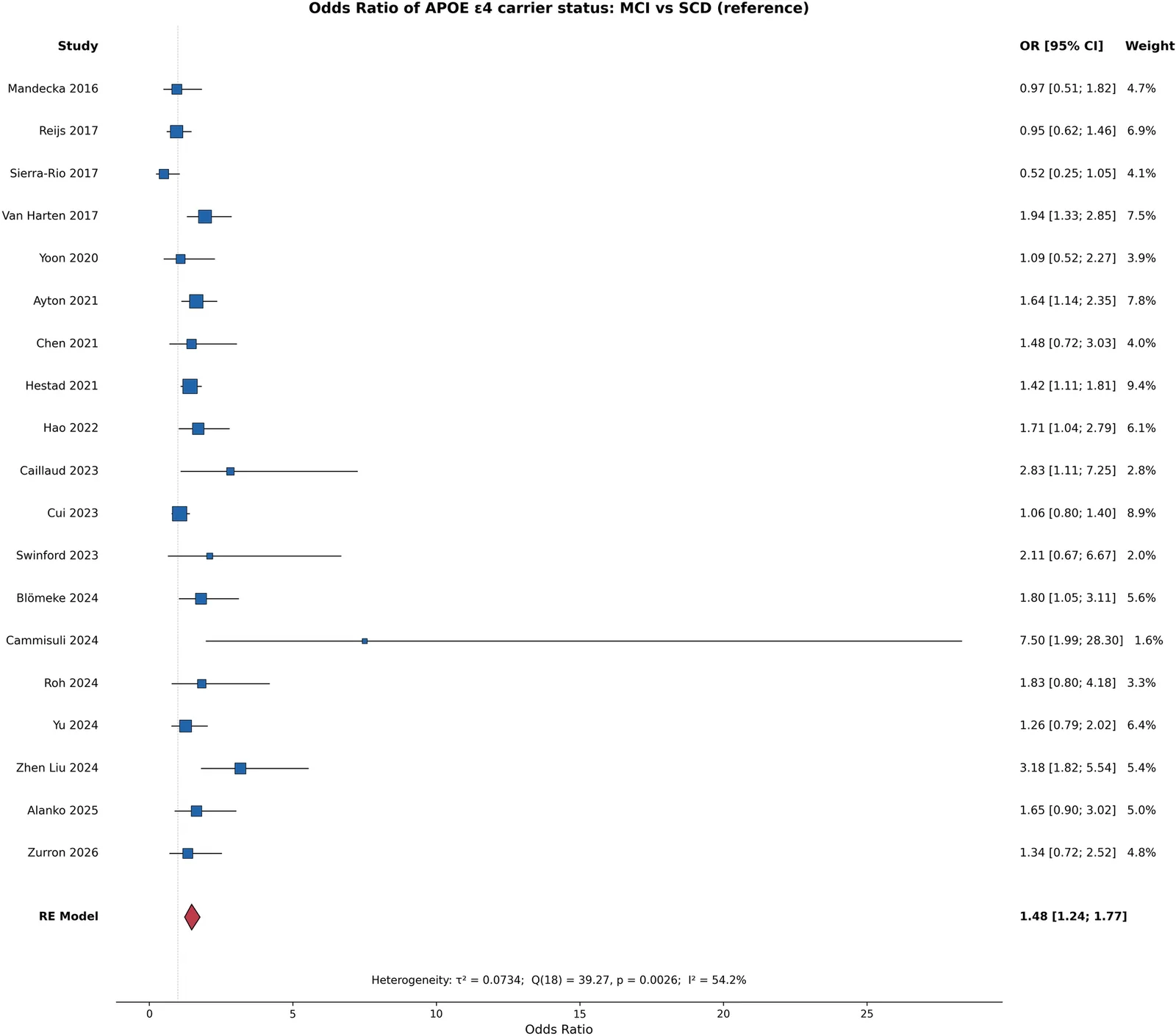
Forest plots of the odds ratios (*ORs*) for APOE ε4 carriership comparing MCI vs SCD (reference). Each square represents the study-specific OR, with the size proportional to the study weight. Horizontal lines indicate 95% confidence intervals. The red diamond represents the pooled OR from the random-effects model (*REML*). The dashed vertical line indicates OR = 1 (no difference). Studies are ordered by year of publication and alphabetically within each year

**Fig. 4:**
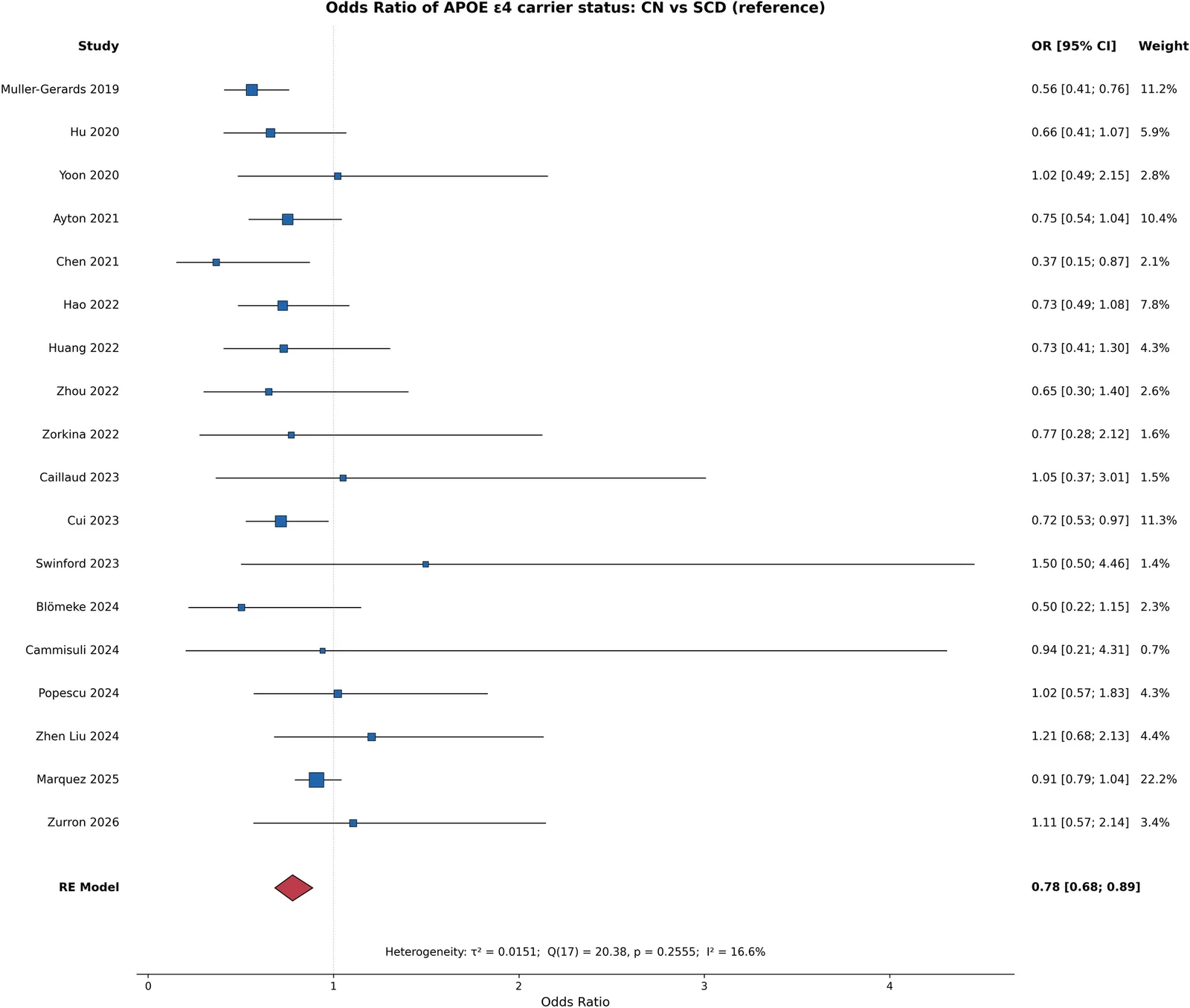
Forest plots of the odds ratios (*ORs*) for APOE ε4 carriership comparing SCD vs cognitively normal individuals (CN, reference group). For ease of interpretation, the OR was inverted (i.e., SCD vs CN) so that ORs greater than 1 consistently indicate a higher prevalence of APOE ε4 carriership in the more impaired group. Each square represents the study-specific OR, with the size proportional to the study weight. Horizontal lines indicate 95% confidence intervals. The red diamond represents the pooled OR from the random-effects model (*REML*). The dashed vertical line indicates OR = 1 (no difference). Studies are ordered by year of publication and alphabetically within each year

Twenty-six studies were included in the multivariate model. The odds of being an APOE ε4 carrier were significantly higher in subjects SCD than in healthy controls (OR = 1.28, 95% CI 1.12–1.46, *p* <0.001); the odds in people with MCI were significantly higher than in those with SCD (OR = 1.48, 95% CI 1.23–1.76, *p* <0.0001,). The pairwise comparisons of the odds of being an APOE ε4 carrier across diagnostic groups are displayed in Figs. 3 and 4. See also Supplementary material Table S4d, e.

### Sensitivity analysis

Thirty-one studies used the SCD *plus* definition and were included in the sensitivity analysis. The prevalence of APOE ε4 carrier status was consistent with that observed in the main analysis.

(29.1%, 95% CI 25.7–32.9%). The mean number of years of education among participants was positively correlated with APOE ε4 carrier status (*β* = 0.17, *p* <0.001), whilst the proportion of female participants was not correlated (*p* = 0.776); however, unlike in the main analysis, the mean age of participants showed no correlation with the prevalence of APOE ε4 carrier status (*p* = 0.624). The results of the multivariate analysis of ORs among the three groups (SCD+, MCI and HC) were similar to those of the main analysis (see Supplementary Material Table S5k–p for further details).

### Risk of bias assessment

Overall, most studies were assessed as having a low or moderate risk of bias, whilst only six studies rated as high risk. Detailed assessments are reported in Supplementary Material (Table S6a).

## Discussion

Genetic risk factors have gained growing interest in the management of dementia as tools for risk stratification, complementing biomarkers in CSF and blood. Indeed, as attention shifts towards the pre-symptomatic stages of cognitive decline, patient selection becomes crucial to focus on at-risk populations and to manage and allocate diagnostic and therapeutic resources appropriately. In this scenario, the APOE gene undoubtedly plays a pivotal role. Indeed, in-vitro and in-vivo studies showed that APOE ε4 promotes the seeding of Aβ peptide, fostering the deposition of fibrils, whilst simultaneously inhibiting their clearance [69, 70].

Moreover, studies using gene silencing have shown that the negative effects of APOE ε4 allele are more pronounced in the earliest phases of plaque deposition. [71] Finally, the role of the APOE ε4 allele is becoming increasingly significant in the therapeutic context as well, as it has been shown to be a reliable predictor of amyloid related imaging abnormalities (ARIA) during therapy with new anti-amyloid monoclonal antibodies; furthermore, it is currently being evaluated as a therapeutic target itself, through techniques, such as gene editing, gene silencing, gene expression regulation and even lifestyle-related strategies. [72, 73] A comprehensive assessment of the prevalence and clinical significance of the APOE ε4 allele in individuals with SCD is, therefore, required, placing this population within the broader AD continuum, to better understand the implications of APOE for disease progression and risk.

In the present review, the prevalence of APOE ε4 allele in SCD was found to be consistently intermediate between CN individuals and subjects with MCI, supporting the view of SCD as a transitional stage. Our findings contradict a previous review, which reported a similar prevalence of APOE ε4 allele in SCD and CN subjects. [13] These conflicting results can be partly explained partially explained by the different definition of SCD used in this study compared with the previous one; indeed, Ali et al. included studies that used earlier definitions, whereas data in this review focused on studies that applied the more stringent criteria of the Subjective Cognitive Decline Initiative working group [5].

However, one of the main findings of this study is considerable heterogeneity observed across the various included studies, which remained high despite the use of meta-regression analysis. This heterogeneity is not surprising, as it reflects similar findings that have already been widely described in relation to MCI and is likely due to several factors. [74]. First, both SCD and MCI are syndromic umbrella terms rather than specific diseases, and they encompass different underlying aetiologies (e.g. vascular, degenerative, inflammatory, etc.). Second, variability in SCD definitions persists despite the introduction of standardised criteria, with differences in recruitment settings (hospital- vs population-based cohorts) and heterogeneity in assessment tools. Third, geographical and ethnic differences may influence APOE ε4 allele distribution, as previously documented in populations with AD and MCI, with lower frequencies in Mediterranean and Asian cohorts compared to populations in Northern European and North America. Finally, methodological heterogeneity (e.g. differences in recruitment strategies, genotyping methods, and sample size) may further contribute to the observed variability.

The meta-regression approach provides further insight into the factors influencing the distribution of the APOE ε4 allele. For example, the negative association observed with age in SCD populations may reflect a survival bias or a more rapid conversion of APOE ε4 carriers to more advanced stages of the disease, thereby reducing their presence in cohorts of older SCD subjects. Conversely, the positive association between SCD and education may reflect selection bias or the influence of cognitive reserve, whereby individuals with higher educational attainment are more likely to report mild cognitive changes at an early stage, even though their objective cognitive performance remains within the normal range.

Another interesting insight emerges from a comparison of the results of cross-sectional and longitudinal studies. Whilst the former show heterogeneous results regarding the association between the APOE ε4 allele and an increase in subjective complaints or mild cognitive deficits, longitudinal studies provide more consistent evidence regarding the clinical relevance of the APOE ε4 allele in SCD, demonstrating that APOE ε4 carriers have a higher risk of conversion to MCI and/or AD dementia, with faster rates of cognitive decline. [50, 51]. Moreover, cross-sectional studies show evidence of associations between CSF/blood biomarkers and APOE status, further confirming the relevance of its biological role in SCD as well. [29, 32, 34, 44] However, this effect is not uniformly observed, reflecting how progression from SCD appears to be influenced by a complex interaction between genetic susceptibility, biomarker status, comorbidities, and lifestyle factors [33, 35, 46] 

A relevant implication of these findings is that the APOE ε4 allele should not be considered in isolation when assessing SCD populations, but should instead be integrated into multimodal risk models that include fluid and imaging biomarkers, as well as clinical and demographic variables, and other genes, such as PER2 and CLOCK. [75] Over time, this approach could lead to personalised care and counselling for at-risk patients. Furthermore, the role of the APOE ε4 allele in modulating the response to interventions and influencing patient behaviour warrants further investigation. Studies examining APOE disclosure suggest that awareness of genetic risk may influence future planning and health-related behaviours, although its psychological impact appears to be generally manageable. [57, 58] These findings highlight the broader clinical and ethical implications of incorporating genetic testing into early-stage cognitive assessment.

This study has several strengths. It includes a large number of studies covering various populations and employs robust meta-analytical techniques, including meta-regression to analyse sources of heterogeneity and multivariate modelling to account for the correlation between pairwise odds ratios sharing a common reference group. Furthermore, the simultaneous comparison across SCD, MCI, and CN groups provides a comprehensive overview of where SCD fits within the APOE ε4–related risk gradient along the AD continuum. The risk of bias was found to be low in many of the assessed domains. Regarding publication bias, the negative intercept of Egger's test indicated that smaller studies tended to report lower APOE ε4 prevalence—a pattern opposite to that expected in the case of classical publication bias, where small studies typically tend to overestimate effects. This finding is more consistent with a well-known methodological artifact of the Egger test when applied to meta-analyses of proportions rather than with true publication bias. This interpretation is further supported by the trim-and-fill analysis, which imputed very few hypothetically missing studies and produced adjusted estimates virtually identical to the original ones, with no change in the direction or statistical significance of any pooled result.

However, several limitations must be considered. First, considerable heterogeneity was observed among the included studies, particularly in the SCD populations, which likely reflects residual variability in diagnostic criteria, recruitment methods (population- vs. hospital-based), and methodological approaches. Although strict inclusion criteria were applied in line with the SCD initiative working group, differences in operationalization of SCD and variability in neuropsychological assessment protocols may have influenced the comparability of the data. Second, potential selection bias cannot be ruled out, as many studies were conducted in memory clinics, potentially enriching the samples with individuals at higher risk of progression to MCI/dementia. Third, most included studies were not specifically designed to estimate the prevalence of APOE ε4 in SCD populations; rather, genotype data were reported as a descriptive sample characteristic, which may limit the precision and comparability of the pooled estimates. Finally, the incomplete reporting of relevant covariates (e.g., comorbidities, lifestyle factors, and other genetic variants) limited our ability to fully explore sources of heterogeneity and potential confounding effects. In addition, meta-regression analyses were based on study-level rather than individual-level data, which limited the precision of these results. Finally, the included studies were conducted in different geographical regions and healthcare systems, which may differ in terms of referral pathways, diagnostic criteria for SCD, and the clinical setting in which APOE genotyping is performed. Although the leave-one-out analysis showed that no individual study disproportionately influenced the pooled estimates, it was not possible to carry out a formal subgroup analysis by geographical region due to the heterogeneous and unbalanced distribution of studies across countries. Consequently, the extent to which regional factors may contribute to the observed heterogeneity between-study remains uncertain.

## Conclusion

The prevalence of APOE ε4 in individuals with SCD is intermediate between cognitively normal individuals and those with MCI, supporting the notion that SCD represents a transitional stage within the AD continuum. However, considerable heterogeneity persists across studies, consistent with previous reports, reflecting residual variability in SCD definitions, recruitment methods, and population characteristics. These findings suggest that APOE ε4 genotyping may contribute to the biological characterisation of populations with SCD and to the identification of individuals at higher risk of progression to MCI/dementia. Future large-scale prospective studies, enrolling individuals with SCD according to current standardised criteria and integrating genetic, biomarker, and clinical data, will be essential to further clarify the role of APOE ε4 in the preclinical stages of AD and to inform personalised risk stratification strategies.

## Supplementary Information

Below is the link to the electronic supplementary material.

Supplementary file1 (DOCX 356 kb)

## Data Availability

The datasets used and/or analysed during the current study are available from the corresponding author upon reasonable request.
