# Supplementary material for: APOE in subjective cognitive decline: a systematic review and meta-analysis
Source: J Neurol. 2026 Aug 30;273(9):556. doi: 10.1007/s00415-026-14077-5 (PMC13526724; doi:10.1007/s00415-026-14077-5)
Supplement: Supplementary file 1 [file 415_2026_14077_MOESM1_ESM.docx]

# Supplementary Material

**Supplementary Table S1. Search strategy**

**SCOPUS**

( TITLE-ABS-KEY ( "subjective cognitive decline" OR "subjective cognitive complaint*" OR "subjective memory complaint*" ) ) AND ( TITLE-ABS-KEY ( APP OR PSEN1 OR PSEN2 OR APOE OR TREM2 OR SORL1 OR ABCA7 OR CLU OR PICALM OR CR1 OR BIN1 OR CD33 OR EPHA1 OR MS4A6A OR CD2AP OR "genetic" OR "gene" ) ) AND NOT TITLE-ABS-KEY ( "review" ) AND ( LIMIT-TO ( DOCTYPE , "ar" ) ) AND ( LIMIT-TO ( EXACTKEYWORD , "Human" ) )

**PubMed**

("subjective cognitive decline"[tiab] OR "subjective cognitive complaint*"[tiab] OR "subjective memory complaint*"[tiab]) AND ( APP[tiab] OR PSEN1[tiab] OR PSEN2[tiab] OR APOE[tiab] OR TREM2[tiab] OR SORL1[tiab] OR ABCA7[tiab] OR CLU[tiab] OR PICALM[tiab] OR CR1[tiab] OR BIN1[tiab] OR CD33[tiab] OR EPHA1[tiab] OR MS4A6A[tiab] OR CD2AP[tiab] OR "genetic" OR "gene") AND (humans[Mesh]) NOT (review[pt])

**Web of Science**

"subjective cognitive decline" OR "subjective cognitive complaint" OR "subjective memory complaint" (Topic) and APP OR PSEN1 OR PSEN2 OR APOE OR TREM2 OR SORL1 OR ABCA7 OR CLU OR PICALM OR CR1 OR BIN1 OR CD33 OR EPHA1 OR MS4A6A OR CD2AP (All Fields)

**Supplementary Table S2. Summary of cross-sectional studies included in the meta-analysis (n = 26)**

| **Study** | **Country** | **Population** | **Key assessment** | **Main findings** | **APOE ε4 effect** |
| --- | --- | --- | --- | --- | --- |
| Alanko et al., 2025 | Sweden | AD spectrum (SCD, MCI, AD dementia) | CSF protein panel (ATN classification) | Reliable differentiation among ATN categories, indicating biological diagnostic signatures | Not assessed |
| Baquero et al., 2024 | Spain | AD-spectrum cohort | APOE genotype | Most non-AD participants were non-ε4 carriers; homozygous ε4 non-AD subjects showed greater cognitive impairment | Significant effect on cognitive performance |
| Blömeke et al., 2024 | Germany | APOE ε4 carriers and non-carriers | Aβ42 oligomers, p-tau | Higher Aβ42 oligomers and shift toward higher p-tau levels in ε4 carriers | Association of genotype with biomarker levels |
| Cammisuli et al., 2024 | Italy | CN, SCD, MCI | Visuospatial processing, APOE | SCD impaired vs CN; APOE ε4 carriers showed worse visuospatial performance, particularly in MCI | Negative effect on cognition |
| Hsieh et al., 2024 | Taiwan | CN, SCD | Plasma Aβ42 | Lower plasma Aβ42 discriminates SCD from CN and predicts cognitive decline | Not reported |
| Roh et al., 2024 | South Korea | AD spectrum (SCD, MCI, AD dementia) | MRI + amyloid PET | White matter patterns classify impairment severity; correlate with blood biomarkers | Not reported |
| Yu et al., 2024 | Australia | SCD, MCI | hs-CRP, cognition | Systemic inflammation linked to worse cognition in SCD, particularly in APOE ε4 non-carrier genotype | Interaction (effect in non-carriers) |
| Cui 2023 | China | CN, SCD, MCI | APOE genotype distribution, neuropsychological battery | APOE ε4 carrier frequency differs across diagnostic groups; higher in MCI than CN and SCD | Risk gradient across diagnostic groups |
| Hong et al., 2023 | China | SCD (amyloid-positive vs amyloid-negative) | APOE genotype, cognition | Amyloid-positive SCD subjects were older and more frequently APOE ε4 carriers | Associated with cerebral amyloidosis |
| Swinford et al., 2023 | USA | SCD without cardiovascular risk factors | Cerebral blood flow (ASL-MRI) | APOE ε4 negatively affects cerebral blood flow in SCD | Negative effect on cerebral perfusion |
| Caillaud et al., 2022 | Canada | SCD | Hippocampal volume | Hippocampal volume predicts associative memory performance; no APOE effect observed | No effect |
| Hao et al., 2022 | China | CN, SCD, MCI | APOE ε4 allele frequency | APOE ε4 frequency increases with the degree of cognitive impairment; correlated with the SCD severity | Positive association with the severity of impairment |
| Huang et al., 2022 | China | CN, SCD | Plasma p-tau181, NfL | Plasma p-tau181 and Nf levels increased in SCD compared to CN | Not the primary target of investigation |
| Moschini et al., 2022 | Italy | SCD | CAG repeat length (HTT gene), personality traits | Longer CAG repeats in the HTT gene are associated with specific personality traits in SCD | Assessed as covariate |
| Zhou et al., 2022 | China | CN, SCD | fMRI functional connectivity | Reduced brain network dynamics in SCD correlated with APOE ε4 allele count | Negative association with network dynamics |
| Ayton et al., 2021 | Sweden | AD spectrum (CN, SCD, MCI, AD dementia) | CSF acute-phase reactant proteins | CSF inflammation markers linked to disease progression, independently of APOE genotype | No independent effect |
| Chen et al., 2021 | China | SCD | Dynamic functional connectivity (fMRI) | Brain network reorganization in SCD; no APOE effect on connectivity patterns | No effect |
| Hestad et al., 2021 | Norway | AD spectrum (CN, SCD, MCI, AD dementia) | Neuropsychological performance | APOE ε4 negatively impacts cognition across all diagnostic stages | Strong negative effect across all stages |
| Hu et al., 2020 | China | CN, SCD | Apolipoprotein B, apolipoprotein A1, CSF biomarkers | Lower apolipoprotein B linked to a more unfavorable AD biomarker profile (especially in SCD), regadless of APOE genotype | Independent effect |
| Lee et al., 2020 | South Korea | SCD (euthyroid subjects) | Thyroid hormones (T3, T4, TSH) | T3 beneficial in APOE ε4 carriers but detrimental in non-carriers | Gene–hormone interaction |
| Yoon et al., 2020 | South Korea | CN, SCD | Balance and mobility assessment | Early motor impairment in SCD; worse in females and APOE ε4 carriers | Negative effect on motor function |
| De Rojas et al., 2018 | Spain | SCD | Plasma Aβ42/40 ratio | Modest association between plasma Aβ42/40 and brain amyloid burden, independently of APOE | Independent of APOE |
| Mandecka et al., 2016 | Europe (multicentre) | SCD, MCI, AD dementia | CSF Aβ, tau | CSF biomarkers linked to impairment severity; APOE ε4 predicts stronger biomarker changes but not cognitive outcomes | Mixed effect (biomarkers but not cognition) |
| Reijs et al., 2017 | Poland | SCD, MCI | Lifestyle factors | Lifestyle had no impact on biomarkers or conversion rates; no interaction with APOE genotype was observed | No effect |
| Zurron et al., 2026 | Spain | CN, SCD, MCI | MRI + plasma biomarkers | Single-domain amnestic MCI and multi-domain amnestic MCI represent two distinct sequential stages; non-amnestic MCI does not appear to be associated with AD pathology; SCD does not carry a higher risk of progression than CN | No differences were found in the distribution of APOE ε4 among the diagnostic groups |
| Lautenschlager et al., 2005 | Australia | Women with SCD or MCI | Homocysteine, APOE genotype | Higher homocysteine levels in MCI; only a trend toward an association with APOE ε4 genotype is observed | Weak or absent |

CN: cognitively normal; SCD: subjective cognitive decline; MCI: mild cognitive impairment; AD: Alzheimer’s disease; APOE: apolipoprotein E; CSF: cerebrospinal fluid; ATN: amyloid/tau/neurodegeneration; Aβ: amyloid beta; p-tau: phosphorylated tau; NfL: neurofilament light chain; GFAP: glial fibrillary acidic protein; hs-CRP: high-sensitivity C-reactive protein; fMRI: functional magnetic resonance imaging; ASL: arterial spin labelling; PET: positron emission tomography; HTT: huntingtin; CAG: cytosine–adenine–guanine.

**Supplementary Table S3. Summary of longitudinal studies included in the meta-analysis (n = 23)**

| **Study** | **Country** | **Population** | **Key assessment** | **Main findings** | **APOE ε4 effect** |
| --- | --- | --- | --- | --- | --- |
| Giacomucci et al., 2025 | Italy | SCD cohort | Gender, cognitive reserve, longitudinal follow-up | Lower cognitive reserve is associated with a higher risk of progression to MCI; women had lower cognitive reserve, but gender was not independently associated with progression | Not the primary factor |
| Marquez et al., 2025 | USA | Hispanic/Latino SCD cohort | p-tau181, NfL, GFAP, Aβ42/40 | Plasma p-tau181, NfL, and GFAP associated with SCD severity; Aβ42/40 ratio and APOE ε4 genotype not associated | No effect |
| Jurasova et al., 2024 | Czech Republic | SCD, MCI, AD dementia | CSF neurogranin | CSF neurogranin lower in SCD/MCI vs AD dementia; higher in APOE ε4 carriers with amnestic MCI | Modulates synaptic biomarker levels |
| Kang et al., 2024 | USA | SCD cohort | SCD-plus criteria, longitudinal follow-up | SCD-plus criteria predict an increased risk of MCI and dementia, independently of APOE ε4 genotype | No independent effect |
| Morrison et al., 2024 | USA | CN, SCD, MCI | Longitudinal cognitive assessment | SCD subjects carrying APOE ε4 show faster cognitive decline over time | APOE ε4 increases decline rate |
| Popescu et al., 2024 | USA | SCD | APOE genotype disclosure, behavioural outcomes | Disclosure of APOE status influenced planning of future directives in SCD individuals | Behavioural impact only |
| Zhen Liu et al., 2024 | China | CN, SCD, MCI, AD dementia | Plasma biomarkers (p-tau181, GFAP), neuroimaging | SCD showed higher p-tau181 and GFAP levels than CN; MCI showed more advanced pathology | Not assessed as primary variable |
| Lee et al., 2023 | USA | Research volunteers | APOE genotype disclosure, psychological outcomes | No increase in psychological distress following disclosure; non-SCD subjects showed better coping and planning | Not assessed as cognitive modifier |
| Tippett et al., 2022 | New Zealand | CN, SCD, MCI, AD dementia | APOE ε4 genotype distribution across stages | APOE ε4 frequency higher in SCD compared to CN, but lower than in MCI | Risk gradient across diagnostic stages |
| Zorkina et al., 2022 | Russia | CN (elderly), SCD | Cognitive training, APOE genotype | Response to cognitive training varies depending on APOE ε4 status in older adults with SCD | Modulates cognitive training response |
| Janssen et al., 2021 | Netherlands | SCD cohort | Amyloid PET positivity | Age, clinic-based setting, and APOE ε4 carrier status are associated with amyloid positivity in SCD | Positive predictor of amyloid positivity |
| Baldacci et al., 2020 | France | SCD cohort | Plasma NfL, tau | Increased plasma NfL levels in APOE ε4 carriers with concurrent amyloid positivity | Interaction with amyloid status |
| Bessi et al., 2020 | Italy | SCD cohort | PER2 C111G polymorphism, cognitive reserve | PER2 polymorphism interacts with cognitive reserve in predicting cognitive trajectories in SCD | Assessed as covariate |
| Mazzeo et al., 2020 | Italy | SCD cohort | Age, APOE genotype, longitudinal follow-up | Patients aged ≥60 years carrying APOE ε4 genotype had an increased risk of conversion to MCI or dementia | Strong predictor of conversion |
| Stockmann et al., 2020 | Netherlands | SCD cohort | Plasma Aβ biomarkers (misfolding, Aβ42/40 ratio) | Aβ misfolding and Aβ42/40 ratio strongly predict progression to MCI/dementia | Markers adjusted for APOE; specific role not described |
| Müller-Gerards et al., 2019 | Germany | CN, SCD | APOE ε4, incident MCI | SCD subjects carrying APOE ε4 genotype had a higher risk of developing incident MCI during the follow-up period | Increased risk of MCI conversion |
| Dubois et al., 2018 | France | Preclinical AD (SCD) | Amyloid biomarkers, longitudinal cognition | Amyloid positivity not associated with short-term cognitive decline; APOE ε4 strongly predicted progression to prodromal AD in participants positive for amyloid β deposition | Risk increasement |
| Fladby et al., 2017 | Norway | At-risk individuals (including SCD) | CSF Aβ, APOE genotype | APOE ε4 more frequent in at-risk groups even in the absence of amyloid positivity | Early risk marker |
| Sierra-Rio et al., 2017 | Spain | SCD, MCI | CSF biomarkers (Aβ42, p-tau) | Pathological CSF Aβ42/p-tau ratio strongly predicts progression to AD dementia | Independent of APOE |
| Van Harten et al., 2017 | Netherlands | SCD, MCI | CSF and plasma apolipoprotein E levels | CSF apolipoprotein E levels predict clinical progression only in ε4 carriers | Genotype-specific effect |
| Wolfsgruber et al., 2016 | Germany | CN, SCD | Clinical progression in stable vs inconsistent SCD | Persistent and worrisome SCD strongly predicted incident AD dementia; inconsistent SCD did not | Independent effect on AD dementia risk |
| Kryscio et al., 2014 | USA | Memory complaints cohort | Subjective memory complaints, neuropathology | Subjective memory complaints linked to increased AD risk; APOE ε4 genotype doubles odds of progression | Strong risk modifier |
| Xu et al., 2023 | China | SCD cohort | Progression to AD or vascular dementia; vascular, psychiatric, sleep, and genetic factors | APOE ε4 carrier status is associated with progression from SCD to AD | Increased AD conversion risk |

CN: cognitively normal; SCD: subjective cognitive decline; MCI: mild cognitive impairment; AD: Alzheimer’s disease; APOE: apolipoprotein E; CSF: cerebrospinal fluid; Aβ: amyloid beta; p-tau: phosphorylated tau; NfL: neurofilament light chain; GFAP: glial fibrillary acidic protein; PET: positron emission tomography; PER2: period circadian regulator 2.

**Supplementary Table S4. Pooled prevalence estimates and pairwise odds ratio comparisons**

Random-effects models with restricted maximum likelihood (REML) estimation. Proportions were logit-transformed before meta-analysis; back-transformed proportions are reported. Odds ratios use SCD as the reference group. Continuity correction of 0.5 applied where needed.

**a. Pooled proportion of APOE ε4 carriers in SCD (k = 49)**

| **Study** | **APOE ε4 carriers** | **Total n** | **Proportion** | **Logit(p)** | **SE** | **95% CI lower** | **95% CI upper** | **Weight (%)** |
| --- | --- | --- | --- | --- | --- | --- | --- | --- |
| Lautenschlager 2005 | 24 | 121 | 0.20 | -1.3967 | 0.2280 | 0.14 | 0.28 | 2.0 |
| Kryscio 2014 | 160 | 531 | 0.30 | -0.8410 | 0.0946 | 0.26 | 0.34 | 2.3 |
| Mandecka 2016 | 28 | 85 | 0.33 | -0.7108 | 0.2308 | 0.24 | 0.44 | 2.0 |
| Wolfsgruber 2016 | 24 | 130 | 0.18 | -1.4854 | 0.2261 | 0.13 | 0.26 | 2.0 |
| Fladby 2017 | 64 | 164 | 0.39 | -0.4463 | 0.1601 | 0.32 | 0.47 | 2.2 |
| Reijs 2017 | 51 | 111 | 0.46 | -0.1625 | 0.1905 | 0.37 | 0.55 | 2.1 |
| Sierra-Rio 2017 | 40 | 55 | 0.73 | 0.9808 | 0.3028 | 0.60 | 0.83 | 1.8 |
| Van Harten 2017 | 86 | 206 | 0.42 | -0.3331 | 0.1413 | 0.35 | 0.49 | 2.2 |
| De Rojas 2018 | 52 | 200 | 0.26 | -1.0460 | 0.1612 | 0.20 | 0.33 | 2.2 |
| Dubois 2018 | 62 | 318 | 0.20 | -1.4180 | 0.1415 | 0.16 | 0.24 | 2.2 |
| Muller-Gerards 2019 | 83 | 292 | 0.28 | -0.9235 | 0.1297 | 0.24 | 0.34 | 2.2 |
| Baldacci 2020 | 62 | 316 | 0.20 | -1.4102 | 0.1417 | 0.16 | 0.24 | 2.2 |
| Bessi 2020 | 14 | 41 | 0.34 | -0.6568 | 0.3293 | 0.21 | 0.50 | 1.7 |
| Hu 2020 | 41 | 219 | 0.19 | -1.4682 | 0.1732 | 0.14 | 0.24 | 2.1 |
| Lee 2020 | 49 | 197 | 0.25 | -1.1054 | 0.1648 | 0.19 | 0.31 | 2.2 |
| Mazzeo 2020 | 42 | 150 | 0.28 | -0.9445 | 0.1818 | 0.21 | 0.36 | 2.1 |
| Stockmann 2020 | 77 | 203 | 0.38 | -0.4925 | 0.1446 | 0.32 | 0.45 | 2.2 |
| Yoon 2020 | 20 | 96 | 0.21 | -1.3350 | 0.2513 | 0.14 | 0.30 | 2.0 |
| Ayton 2021 | 82 | 210 | 0.39 | -0.4453 | 0.1414 | 0.33 | 0.46 | 2.2 |
| Chen 2021 | 26 | 81 | 0.32 | -0.7492 | 0.2380 | 0.23 | 0.43 | 2.0 |
| Hestad 2021 | 174 | 425 | 0.41 | -0.3664 | 0.0986 | 0.36 | 0.46 | 2.3 |
| Janssen 2021 | 464 | 1640 | 0.28 | -0.9300 | 0.0548 | 0.26 | 0.31 | 2.3 |
| Hao 2022 | 80 | 298 | 0.27 | -1.0025 | 0.1307 | 0.22 | 0.32 | 2.2 |
| Huang 2022 | 33 | 111 | 0.30 | -0.8602 | 0.2077 | 0.22 | 0.39 | 2.1 |
| Moschini 2022 | 17 | 54 | 0.31 | -0.7777 | 0.2930 | 0.21 | 0.45 | 1.8 |
| Tippett 2022 | 27 | 60 | 0.45 | -0.2007 | 0.2595 | 0.33 | 0.58 | 1.9 |
| Zhou 2022 | 32 | 95 | 0.34 | -0.6774 | 0.2171 | 0.25 | 0.44 | 2.0 |
| Zorkina 2022 | 24 | 125 | 0.19 | -1.4371 | 0.2271 | 0.13 | 0.27 | 2.0 |
| Caillaud 2023 | 13 | 66 | 0.20 | -1.4053 | 0.3095 | 0.12 | 0.31 | 1.8 |
| Cui 2023 | 137 | 578 | 0.24 | -1.1691 | 0.0978 | 0.20 | 0.27 | 2.3 |
| Hong 2023 | 21 | 120 | 0.17 | -1.5506 | 0.2402 | 0.12 | 0.25 | 2.0 |
| Lee 2023 | 37 | 82 | 0.45 | -0.1957 | 0.2219 | 0.35 | 0.56 | 2.0 |
| Swinford 2023 | 8 | 24 | 0.33 | -0.6931 | 0.4330 | 0.18 | 0.54 | 1.5 |
| Xu 2023 | 118 | 997 | 0.12 | -2.0081 | 0.0980 | 0.10 | 0.14 | 2.3 |
| Baquero 2024 | 19 | 165 | 0.12 | -2.0392 | 0.2439 | 0.07 | 0.17 | 2.0 |
| Blömeke 2024 | 46 | 146 | 0.32 | -0.7765 | 0.1782 | 0.24 | 0.39 | 2.1 |
| Cammisuli 2024 | 5 | 23 | 0.22 | -1.2809 | 0.5055 | 0.09 | 0.43 | 1.3 |
| Hsieh 2024 | 6 | 38 | 0.16 | -1.6740 | 0.4449 | 0.07 | 0.31 | 1.4 |
| Jurasova 2024 | 29 | 33 | 0.88 | 1.9810 | 0.5334 | 0.72 | 0.95 | 1.2 |
| Kang 2024 | 237 | 1034 | 0.23 | -1.2128 | 0.0740 | 0.20 | 0.26 | 2.3 |
| Morrison 2024 | 189 | 768 | 0.25 | -1.1196 | 0.0838 | 0.22 | 0.28 | 2.3 |
| Popescu 2024 | 35 | 82 | 0.43 | -0.2948 | 0.2233 | 0.32 | 0.54 | 2.0 |
| Roh 2024 | 7 | 49 | 0.14 | -1.7918 | 0.4082 | 0.07 | 0.27 | 1.5 |
| Yu 2024 | 46 | 133 | 0.35 | -0.6373 | 0.1823 | 0.27 | 0.43 | 2.1 |
| Zhen Liu 2024 | 36 | 207 | 0.17 | -1.5581 | 0.1834 | 0.13 | 0.23 | 2.1 |
| Alanko 2025 | 49 | 151 | 0.32 | -0.7332 | 0.1738 | 0.25 | 0.40 | 2.1 |
| Giacomucci 2025 | 123 | 440 | 0.28 | -0.9467 | 0.1062 | 0.24 | 0.32 | 2.3 |
| Marquez 2025 | 852 | 3837 | 0.22 | -1.2538 | 0.0388 | 0.21 | 0.24 | 2.4 |
| Zurron 2026 | 23 | 122 | 0.19 | -1.4596 | 0.2315 | 0.13 | 0.27 | 2.0 |
| **RE model (pooled)** |  |  | **0.28** |  |  | **0.25** | **0.32** |  |

Heterogeneity: τ² = 0.3012; Q(48) = 488.2, p < 0.0001; I² = 90.2%. Test for overall effect: z = -10.9863, p < 0.0001.

**b. Pooled proportion of APOE ε4 carriers in CN (k = 18)**

| **Study** | **APOE ε4 carriers** | **Total n** | **Proportion** | **Logit(p)** | **SE** | **95% CI lower** | **95% CI upper** | **Weight (%)** |
| --- | --- | --- | --- | --- | --- | --- | --- | --- |
| Muller-Gerards 2019 | 165 | 906 | 0.18 | -1.5021 | 0.0861 | 0.16 | 0.21 | 7.8 |
| Hu 2020 | 38 | 288 | 0.13 | -1.8839 | 0.1741 | 0.10 | 0.18 | 6.8 |
| Yoon 2020 | 15 | 71 | 0.21 | -1.3173 | 0.2907 | 0.13 | 0.32 | 5.2 |
| Ayton 2021 | 200 | 614 | 0.33 | -0.7275 | 0.0861 | 0.29 | 0.36 | 7.8 |
| Chen 2021 | 8 | 56 | 0.14 | -1.7918 | 0.3819 | 0.07 | 0.26 | 4.1 |
| Hao 2022 | 51 | 243 | 0.21 | -1.3257 | 0.1575 | 0.16 | 0.27 | 7.0 |
| Huang 2022 | 29 | 123 | 0.24 | -1.1760 | 0.2124 | 0.17 | 0.32 | 6.3 |
| Zhou 2022 | 12 | 49 | 0.24 | -1.1260 | 0.3322 | 0.14 | 0.38 | 4.7 |
| Zorkina 2022 | 5 | 34 | 0.15 | -1.7579 | 0.4842 | 0.06 | 0.31 | 3.1 |
| Caillaud 2023 | 6 | 30 | 0.20 | -1.3863 | 0.4564 | 0.09 | 0.38 | 3.4 |
| Cui 2023 | 84 | 462 | 0.18 | -1.5041 | 0.1206 | 0.15 | 0.22 | 7.5 |
| Swinford 2023 | 13 | 30 | 0.43 | -0.2683 | 0.3684 | 0.27 | 0.61 | 4.2 |
| Blömeke 2024 | 8 | 44 | 0.18 | -1.5041 | 0.3909 | 0.09 | 0.32 | 4.0 |
| Cammisuli 2024 | 3 | 15 | 0.20 | -1.3863 | 0.6455 | 0.07 | 0.47 | 2.1 |
| Popescu 2024 | 45 | 104 | 0.43 | -0.2709 | 0.1979 | 0.34 | 0.53 | 6.5 |
| Zhen Liu 2024 | 24 | 119 | 0.20 | -1.3758 | 0.2285 | 0.14 | 0.28 | 6.0 |
| Marquez 2025 | 386 | 1875 | 0.21 | -1.3500 | 0.0571 | 0.19 | 0.22 | 8.0 |
| Zurron 2026 | 20 | 98 | 0.20 | -1.3610 | 0.2506 | 0.14 | 0.30 | 5.7 |
| **RE model (pooled)** |  |  | **0.22** |  |  | **0.19** | **0.26** |  |

Heterogeneity: τ² = 0.144; Q(17) = 102.19, p < 0.0001; I² = 83.4%. Test for overall effect: z = -11.609, p < 0.0001.

**c. Pooled proportion of APOE ε4 carriers in MCI (k = 19)**

| **Study** | **APOE ε4 carriers** | **Total n** | **Proportion** | **Logit(p)** | **SE** | **95% CI lower** | **95% CI upper** | **Weight (%)** |
| --- | --- | --- | --- | --- | --- | --- | --- | --- |
| Mandecka 2016 | 28 | 87 | 0.32 | -0.7453 | 0.2295 | 0.23 | 0.43 | 5.3 |
| Reijs 2017 | 158 | 353 | 0.45 | -0.2104 | 0.1070 | 0.40 | 0.50 | 6.1 |
| Sierra-Rio 2017 | 54 | 94 | 0.57 | 0.3001 | 0.2086 | 0.47 | 0.67 | 5.4 |
| Van Harten 2017 | 130 | 223 | 0.58 | 0.3349 | 0.1358 | 0.52 | 0.65 | 6.0 |
| Yoon 2020 | 16 | 72 | 0.22 | -1.2528 | 0.2835 | 0.14 | 0.33 | 4.8 |
| Ayton 2021 | 144 | 281 | 0.51 | 0.0498 | 0.1193 | 0.45 | 0.57 | 6.1 |
| Chen 2021 | 21 | 51 | 0.41 | -0.3567 | 0.2845 | 0.29 | 0.55 | 4.8 |
| Hestad 2021 | 332 | 669 | 0.50 | -0.0149 | 0.0773 | 0.46 | 0.53 | 6.3 |
| Hao 2022 | 35 | 91 | 0.38 | -0.4700 | 0.2155 | 0.29 | 0.49 | 5.4 |
| Caillaud 2023 | 12 | 29 | 0.41 | -0.3483 | 0.3770 | 0.25 | 0.60 | 4.0 |
| Cui 2023 | 118 | 478 | 0.25 | -1.1154 | 0.1061 | 0.21 | 0.29 | 6.1 |
| Swinford 2023 | 12 | 23 | 0.52 | 0.0870 | 0.4174 | 0.32 | 0.71 | 3.7 |
| Blömeke 2024 | 40 | 88 | 0.45 | -0.1823 | 0.2141 | 0.35 | 0.56 | 5.4 |
| Cammisuli 2024 | 14 | 20 | 0.70 | 0.8473 | 0.4880 | 0.47 | 0.86 | 3.2 |
| Roh 2024 | 63 | 259 | 0.24 | -1.1350 | 0.1448 | 0.19 | 0.30 | 5.9 |
| Yu 2024 | 67 | 167 | 0.40 | -0.4005 | 0.1579 | 0.33 | 0.48 | 5.8 |
| Zhen Liu 2024 | 35 | 87 | 0.40 | -0.3959 | 0.2186 | 0.30 | 0.51 | 5.3 |
| Alanko 2025 | 27 | 61 | 0.44 | -0.2305 | 0.2578 | 0.32 | 0.57 | 5.0 |
| Zurron 2026 | 26 | 109 | 0.24 | -1.1607 | 0.2247 | 0.17 | 0.33 | 5.3 |
| **RE model (pooled)** |  |  | **0.41** |  |  | **0.35** | **0.47** |  |

Heterogeneity: τ² = 0.2372; Q(18) = 182.49, p < 0.0001; I² = 90.1%. Test for overall effect: z = -2.9386, p 0.0033.

**d. Odds ratio of APOE ε4 carriership: CN vs SCD (k = 18)**

| **Study** | **log(OR)** | **SE** | **OR** | **95% CI lower** | **95% CI upper** | **Weight (%)** |
| --- | --- | --- | --- | --- | --- | --- |
| Muller-Gerards 2019 | -0.5798 | 0.1554 | 0.56 | 0.41 | 0.76 | 11.2 |
| Hu 2020 | -0.4139 | 0.2443 | 0.66 | 0.41 | 1.07 | 5.9 |
| Yoon 2020 | 0.0235 | 0.3796 | 1.02 | 0.49 | 2.15 | 2.8 |
| Ayton 2021 | -0.2831 | 0.1652 | 0.75 | 0.55 | 1.04 | 10.4 |
| Chen 2021 | -1.0023 | 0.4405 | 0.37 | 0.15 | 0.87 | 2.1 |
| Hao 2022 | -0.3200 | 0.2040 | 0.73 | 0.49 | 1.08 | 7.8 |
| Huang 2022 | -0.3127 | 0.2951 | 0.73 | 0.41 | 1.30 | 4.3 |
| Zhou 2022 | -0.4288 | 0.3914 | 0.65 | 0.30 | 1.40 | 2.6 |
| Zorkina 2022 | -0.2583 | 0.5161 | 0.77 | 0.28 | 2.12 | 1.6 |
| Caillaud 2023 | 0.0501 | 0.5361 | 1.05 | 0.37 | 3.01 | 1.5 |
| Cui 2023 | -0.3329 | 0.1550 | 0.72 | 0.53 | 0.97 | 11.3 |
| Swinford 2023 | 0.4038 | 0.5563 | 1.50 | 0.50 | 4.46 | 1.4 |
| Blömeke 2024 | -0.6865 | 0.4201 | 0.50 | 0.22 | 1.15 | 2.3 |
| Cammisuli 2024 | -0.0599 | 0.7756 | 0.94 | 0.21 | 4.31 | 0.7 |
| Popescu 2024 | 0.0229 | 0.2967 | 1.02 | 0.57 | 1.83 | 4.3 |
| Zhen Liu 2024 | 0.1868 | 0.2907 | 1.21 | 0.68 | 2.13 | 4.4 |
| Marquez 2025 | -0.0957 | 0.0690 | 0.91 | 0.79 | 1.04 | 22.1 |
| Zurron 2026 | 0.1005 | 0.3378 | 1.11 | 0.57 | 2.14 | 3.4 |
| **RE model (pooled)** |  |  | **0.78** | **0.68** | **0.89** |  |

Heterogeneity: τ² = 0.0151; Q(17) = 20.38, p 0.2555; I² = 16.6%. Test for overall effect: z = -3.7505, p 0.0002.

**e. Odds ratio of APOE ε4 carriership: MCI vs SCD (k = 19)**

| **Study** | **log(OR)** | **SE** | **OR** | **95% CI lower** | **95% CI upper** | **Weight (%)** |
| --- | --- | --- | --- | --- | --- | --- |
| Mandecka 2016 | -0.0342 | 0.3231 | 0.97 | 0.51 | 1.82 | 4.7 |
| Reijs 2017 | -0.0487 | 0.2176 | 0.95 | 0.62 | 1.46 | 6.9 |
| Sierra-Rio 2017 | -0.6636 | 0.3637 | 0.52 | 0.25 | 1.05 | 4.1 |
| Van Harten 2017 | 0.6649 | 0.1955 | 1.94 | 1.33 | 2.85 | 7.5 |
| Yoon 2020 | 0.0860 | 0.3744 | 1.09 | 0.52 | 2.27 | 3.9 |
| Ayton 2021 | 0.4928 | 0.1847 | 1.64 | 1.14 | 2.35 | 7.8 |
| Chen 2021 | 0.3896 | 0.3675 | 1.48 | 0.72 | 3.03 | 4.0 |
| Hestad 2021 | 0.3506 | 0.1252 | 1.42 | 1.11 | 1.81 | 9.4 |
| Hao 2022 | 0.5338 | 0.2507 | 1.71 | 1.04 | 2.79 | 6.1 |
| Caillaud 2023 | 1.0405 | 0.4795 | 2.83 | 1.11 | 7.25 | 2.8 |
| Cui 2023 | 0.0540 | 0.1441 | 1.06 | 0.80 | 1.40 | 8.9 |
| Swinford 2023 | 0.7467 | 0.5875 | 2.11 | 0.67 | 6.67 | 2.0 |
| Blömeke 2024 | 0.5904 | 0.2771 | 1.80 | 1.05 | 3.11 | 5.6 |
| Cammisuli 2024 | 2.0154 | 0.6773 | 7.50 | 1.99 | 28.30 | 1.6 |
| Roh 2024 | 0.6050 | 0.4215 | 1.83 | 0.80 | 4.18 | 3.3 |
| Yu 2024 | 0.2342 | 0.2402 | 1.26 | 0.79 | 2.02 | 6.4 |
| Zhen Liu 2024 | 1.1560 | 0.2836 | 3.18 | 1.82 | 5.54 | 5.4 |
| Alanko 2025 | 0.5011 | 0.3087 | 1.65 | 0.90 | 3.02 | 5.0 |
| Zurron 2026 | 0.2955 | 0.3199 | 1.34 | 0.72 | 2.52 | 4.8 |
| **RE model (pooled)** |  |  | **1.48** | **1.24** | **1.77** |  |

Heterogeneity: τ² = 0.0734; Q(18) = 39.27, p 0.0026; I² = 54.2%. Test for overall effect: z = 4.2706, p < 0.0001.

SCD: subjective cognitive decline; CN: cognitively normal; MCI: mild cognitive impairment; APOE: apolipoprotein E; OR: odds ratio; SE: standard error; CI: confidence interval; RE: random effects; REML: restricted maximum likelihood; p: proportion; τ²: between-study variance; I²: percentage of variability due to heterogeneity.

**Supplementary Table S5. Results of meta-regression models**

**a. Meta-regression for participants’ mean age in SCD**

|  | **Estimate** | **SE** | **95% CI** | **p** |
| --- | --- | --- | --- | --- |
| Intercept | 1.72 | 1.31 | -0.85, 4.29 | 0.189 |
| Covariate | -0.04 | 0.02 | -0.08, 0.00 | 0.014 |

SE: standard error; CI: confidence interval

**b. Meta-regression for participants’ mean education years in SCD**

|  | **Estimate** | **SE** | **95% CI** | **p** |
| --- | --- | --- | --- | --- |
| Intercept | -2.24 | 0.53 | -3.29, -1.19 | 0.001 |
| Covariate | 0.09 | 0.04 | 0.01, 0.17 | 0.020 |

SE: standard error; CI: confidence interval

**c. Meta-regression for female participants’ proportion (as categorical variable) in SCD**

|  | **Estimate** | **SE** | **95% CI** | **p** |
| --- | --- | --- | --- | --- |
| Intercept | -0.62 | 0.21 | -1.03, -0.20 | 0.003 |
| Covariate | -0.33 | 0.23 | -0.79, 0.12 | 0.142 |

SE: standard error; CI: confidence interval

**d. Meta-regression for participants’ mean age in MCI**

|  | **Estimate** | **SE** | **95% CI** | **p** |
| --- | --- | --- | --- | --- |
| Intercept | -0.26 | 0.59 | -1.41, 0.89 | 0.659 |
| Covariate | 0.00 | 0.01 | -0.02, 0.02 | 0.945 |

SE: standard error; CI: confidence interval

**e. Meta-regression for participants’ mean education years in MCI**

|  | **Estimate** | **SE** | **95% CI** | **p** |
| --- | --- | --- | --- | --- |
| Intercept | -1.42 | 0.50 | -2.41, -0.44 | 0.005 |
| Covariate | 0.08 | 0.04 | -0.01, 0.17 | 0.098 |

SE: standard error; CI: confidence interval

**f. Meta-regression for female participants’ proportion (as categorical variable) in MCI**

|  | **Estimate** | **SE** | **95% CI** | **p** |
| --- | --- | --- | --- | --- |
| Intercept | -0.15 | 0.15 | -0.44, 0.14 | 0.317 |
| Covariate | -0.44 | 0.23 | -0.89, 0.01 | 0.053 |

SE: standard error; CI: confidence interval

**g. Meta-regression for participants’ mean age in healthy controls**

|  | **Estimate** | **SE** | **95% CI** | **p** |
| --- | --- | --- | --- | --- |
| Intercept | -4.92 | 1.07 | -7.02, -2.82 | 0.001 |
| Covariate | 0.05 | 0.02 | 0.01, 0.09 | 0.007 |

SE: standard error; CI: confidence interval

**h. Meta-regression for participants’ mean education years in healthy controls**

|  | **Estimate** | **SE** | **95% CI** | **p** |
| --- | --- | --- | --- | --- |
| Intercept | -3.62 | 0.52 | -4.65, -2.60 | 0.001 |
| Covariate | 0.12 | 0.04 | 0.04, 0.20 | 0.002 |

SE: standard error; CI: confidence interval

**i. Meta-regression for female participants’ proportion (as categorical variable) in healthy controls**

|  | **Estimate** | **SE** | **95% CI** | **p** |
| --- | --- | --- | --- | --- |
| Intercept | -1.77 | 0.31 | -2.37, -1.17 | 0.001 |
| Covariate | 0.47 | 0.26 | -0.03, 0.98 | 0.067 |

SE: standard error; CI: confidence interval

**j. Comparison of the odds of being an APOE ε4 carrier between HC, MCI and SCD**

| **Comparison** | **OR** | **95% CI** | **p** |
| --- | --- | --- | --- |
| HC vs SCD | 0.78 | 0.68–0.89 | <0.001 |
| MCI vs SCD | 1.48 | 1.24–1.77 | <0.001 |

**k. Pooled proportion of APOE ε4 carriers in SCD-plus studies (k = 31)**

| **Statistic** | **Value** |
| --- | --- |
| Pooled proportion | 0.2901 |
| 95% CI | 0.2570–0.3289 |
| Z | -6.9704 |
| p-value | <0.001 |
| τ² | 0.3470 |
| Q(30) | 227.50 |
| Q p-value | <0.001 |
| I² | 86.8% |

**l. Meta-regression for participants’ mean age in SCD-plus studies**

k = 29

|  | **Estimate** | **SE** | **95% CI** | **p** |
| --- | --- | --- | --- | --- |
| Intercept | 0.05 | 1.95 | -3.77, 3.88 | 0.978 |
| Covariate | -0.01 | 0.04 | -0.0072, 0.043 | 0.624 |

**m. Meta-regression for participants’ mean education years in SCD-plus studies**

k = 23

|  | **Estimate** | **SE** | **95% CI** | **P** |
| --- | --- | --- | --- | --- |
| Intercept | -3.01 | 0.58 | -4.16, -1.87 | <0.001 |
| Covariate | 0.17 | 0.04 | 0.07, 0.25 | <0.001 |

**n. Meta-regression for female participants’ proportion (as categorical variable) in SCD-plus studies**

k = 31

|  | **Estimate** | **SE** | **95% CI** | **P** |
| --- | --- | --- | --- | --- |
| Intercept | -0.81 | 0.25 | -1.30, -0.34 | <0.001 |
| Covariate | -0.08 | 0.27 | -0.06, 0.45 | 0.776 |

**o. Odds ratio of APOE ε4 carriership: SCD-plus vs CN**

| **Statistic** | **Value** |
| --- | --- |
| Pooled OR | 1.41 |
| 95% CI | 1.23–1.63 |
| Z | 4.9062 |
| p-value | <0.001 |
| τ² | 0.0000 |
| Q(12) | 10.00 |
| Q p-value | 0.616 |
| I² | 0.0% |
| K | 13 |

**p. Comparison of the odds of being an APOE ε4 carrier between HC, MCI and SCD-plus**

| **Comparison** | **OR** | **95%CI** | **p** |
| --- | --- | --- | --- |
| HC vs SCD | 0.76 | 0.68-0.87 | <0.01 |
| 95% CI | 1.58 | 1.25-1.99 | <0.001 |

SCD: subjective cognitive decline; CN: cognitively normal; MCI: mild cognitive impairment; OR: odds ratio; CI: confidence interval

**Supplementary Table S6. Publication bias assessment**

**a. JBI Critical Appraisal Checklist for Prevalence Studies**

|  | **Author & Year** | **Study Design** | **Q1** | **Q2** | **Q3** | **Q4** | **Q5** | **Q6** | **Q7** | **Q8** | **Q9** | **Score (/9)** | **Risk of Bias** |
| --- | --- | --- | --- | --- | --- | --- | --- | --- | --- | --- | --- | --- | --- |
| 1 | **Alanko 2025** | Cross-sectional | U | Y | U | U | NA | NA | NA | Y | Y | 3 | High |
| 2 | **Giacomucci 2025** | Longitudinal prospective | Y | Y | U | U | NA | NA | Y | N | Y | 4 | Moderate |
| 3 | **Marquez 2025** | Cross-sectional | Y | Y | N | Y | NA | NA | Y | Y | Y | 6 | Moderate |
| 4 | **Baquero 2024** | Longitudinal prospective | Y | Y | Y | N | NA | NA | Y | Y | Y | 6 | Moderate |
| 5 | **Blömeke 2024** | Cross-sectional | U | U | U | U | NA | NA | Y | Y | Y | 3 | High |
| 6 | **Cammisuli 2024** | Cross-sectional | Y | N | Y | Y | NA | NA | Y | Y | Y | 6 | Moderate |
| 7 | **Hsieh 2024** | Cross-sectional | Y | Y | N | U | NA | NA | Y | Y | NA | 4 | Moderate |
| 8 | **Jurasova 2024** | Cross-sectional | Y | U | Y | Y | NA | NA | Y | Y | Y | 6 | Moderate |
| 9 | **Kang 2024** | Longitudinal prospective | Y | Y | Y | Y | NA | NA | Y | Y | Y | 7 | Low |
| 10 | **Morrison 2024** | Longitudinal prospective | U | Y | U | U | NA | NA | Y | Y | Y | 4 | Moderate |
| 11 | **Popescu 2024** | Longitudinal prospective | Y | Y | U | U | NA | NA | U | U | Y | 3 | High |
| 12 | **Roh 2024** | Longitudinal prospective | U | Y | N | U | NA | NA | U | U | Y | 2 | High |
| 13 | **Swinford 2023** | Cross-sectional | U | Y | N | U | NA | NA | U | U | Y | 2 | High |
| 14 | **Yu 2024** | Cross-sectional | U | Y | Y | Y | NA | NA | Y | U | Y | 5 | Moderate |
| 15 | **Zhen Liu 2024** | Longitudinal prospective | Y | Y | U | U | NA | NA | Y | Y | Y | 5 | Moderate |
| 16 | **Caillaud 2023** | Cross-sectional | Y | Y | Y | N | NA | NA | U | Y | Y | 5 | Moderate |
| 17 | **Cui 2023** | Cross-sectional | Y | Y | U | U | NA | NA | Y | Y | Y | 5 | Moderate |
| 18 | **Hong 2023** | Longitudinal prospective | Y | U | U | U | NA | NA | Y | Y | Y | 4 | Moderate |
| 19 | **Lee 2023** | Longitudinal prospective | Y | Y | N | N | NA | NA | Y | Y | Y | 5 | Moderate |
| 20 | **Hao 2022** | Cross-sectional | Y | Y | Y | Y | NA | NA | Y | Y | Y | 7 | Low |
| 21 | **Huang 2022** | Cross-sectional | Y | Y | Y | Y | NA | NA | Y | Y | Y | 7 | Low |
| 22 | **Moschini 2022** | Cross-sectional | Y | Y | Y | Y | NA | NA | Y | Y | Y | 7 | Low |
| 23 | **Tippett 2022** | Longitudinal prospective | Y | Y | Y | Y | NA | NA | Y | Y | Y | 7 | Low |
| 24 | **Zhou 2022** | Cross-sectional | U | U | U | U | NA | NA | Y | Y | Y | 3 | High |
| 25 | **Zorkina 2022** | Longitudinal prospective | Y | Y | Y | Y | NA | NA | Y | Y | Y | 7 | Low |
| 26 | **Ayton 2021** | Cross-sectional | U | Y | U | U | NA | NA | Y | Y | Y | 4 | Moderate |
| 27 | **Chen 2021** | Cross-sectional | Y | Y | Y | Y | NA | NA | Y | Y | Y | 7 | Low |
| 28 | **Hestad 2021** | Cross-sectional | Y | Y | Y | Y | NA | NA | Y | Y | Y | 7 | Low |
| 29 | **Janssen 2021** | Cross-sectional | Y | Y | Y | Y | NA | NA | Y | Y | Y | 7 | Low |
| 30 | **Baldacci 2020** | Longitudinal prospective | Y | U | U | U | NA | NA | Y | Y | Y | 4 | Moderate |
| 31 | **Bessi 2020** | Longitudinal prospective | Y | Y | Y | Y | NA | NA | N | N | Y | 5 | Moderate |
| 32 | **Hu 2020** | Cross-sectional | Y | Y | Y | Y | NA | NA | Y | Y | Y | 7 | Low |
| 33 | **Lee 2020** | Cross-sectional | Y | Y | Y | Y | NA | NA | Y | Y | Y | 7 | Low |
| 34 | **Mazzeo 2020** | Longitudinal prospective | Y | Y | Y | Y | NA | NA | N | N | Y | 5 | Moderate |
| 35 | **Stockmann 2020** | Longitudinal prospective | Y | Y | Y | Y | NA | NA | N | N | Y | 5 | Moderate |
| 36 | **Yoon 2020** | Longitudinal prospective | Y | Y | Y | Y | NA | NA | N | N | Y | 5 | Moderate |
| 37 | **De Rojas 2018** | Cross-sectional | Y | Y | Y | Y | NA | NA | Y | Y | Y | 7 | Low |
| 38 | **Dubois 2018** | Longitudinal prospective | U | Y | Y | Y | NA | NA | Y | Y | Y | 6 | Moderate |
| 39 | **Fladby 2017** | Longitudinal prospective | Y | Y | Y | Y | NA | NA | Y | Y | Y | 7 | Low |
| 40 | **Reijs 2017** | Cross-sectional | U | Y | Y | U | NA | NA | Y | Y | Y | 5 | Moderate |
| 41 | **Sierra-Rio 2017** | Longitudinal prospective | U | Y | Y | U | NA | NA | Y | Y | Y | 5 | Moderate |
| 42 | **Van Harten 2017** | Longitudinal prospective | U | U | Y | U | NA | NA | Y | Y | Y | 4 | Moderate |
| 43 | **Mandecka 2016** | Cross-sectional | Y | U | Y | Y | NA | NA | Y | Y | Y | 6 | Moderate |
| 44 | **Kryscio 2014** | Longitudinal prospective | U | Y | Y | U | NA | NA | Y | Y | Y | 5 | Moderate |
| 45 | **Lautenschlager 2005** | Longitudinal prospective | U | Y | Y | U | NA | NA | Y | Y | Y | 5 | Moderate |
| 46 | **Muller-Gerards 2019** | Longitudinal prospective | U | Y | Y | U | NA | NA | U | Y | Y | 4 | Moderate |
| 47 | **Zurron 2026** | Longitudinal prospective | Y | Y | Y | Y | NA | NA | Y | Y | Y | 7 | Low |
| 48 | **Wolfsgruber 2016** | Longitudinal prospective | Y | Y | Y | Y | NA | NA | Y | Y | Y | 7 | Low |
| 49 | **Xu 2023** | Longitudinal prospective | Y | Y | Y | Y | NA | NA | Y | Y | Y | 7 | Low |

**Y: Yes; N: No; NA: Not applicable**

**Q1:** Was the sample frame appropriate to address the target population?

**Q2:** Were study participants sampled in an appropriate way?

**Q3:** Was the sample size adequate?

**Q4:** Were the study subjects and the setting described in detail?

**Q5:** Was the data analysis conducted with sufficient coverage of the identified sample?

**Q6:** Were valid methods used for the identification of the condition?

**Q7:** Was the condition measured in a standard, reliable way for all participants?

**Q8:** Was there appropriate statistical analysis?

**Q9:** Was the response rate adequate, and if not, was the low response rate managed appropriately?

**b. Leave-one-out sensitivity analysis and influence diagnostics**

Each study was sequentially removed from the meta-analysis, and the model was re-fitted on the remaining k−1 studies. A study was considered influential when at least one of the following criteria was met: (i) |DFFITS| > 3√(p/(k−p)); (ii) Cook’s distance exceeding the 50th percentile of a χ² distribution with p degrees of freedom; (iii) hat value > 3(p/k); (iv) |DFBETAS| > 1.

In the SCD group (k=49), sequentially omitting each study produced pooled proportions ranging from 0.276 to 0.288. Two studies exceeded the DFFITS thresholds: Sierra-Rio 2017 and Jurasova 2024, both characterised by unusually high APOE ε4 proportions (73% and 88%, respectively); however, their removal affected the pooled estimate by less than one percentage point. In the CN group (k=18), Popescu 2024 was flagged as influential by DFFITS; its omission reduced the pooled proportion from 0.221 to 0.209, without changing the overall interpretation. No influential studies were detected in the MCI group (k=19). Regarding the pairwise comparisons, the pooled OR for CN vs SCD remained significantly below 1 across all leave-one-out iterations. Two studies were flagged as influential: Muller-Gerards 2019, whose removal increased the pooled OR to 0.827 and reduced I² from 16.6% to 0.0%, and Marquez 2025. For MCI vs SCD, the pooled OR remained consistently above 1, and no study met any of the influence criteria. Overall, no single study altered the direction or statistical significance of any pooled estimate, supporting the robustness of the findings despite the heterogeneity in study designs. Egger's regression test indicated significant funnel plot asymmetry for the proportion of APOE ε4 carriers in SCD (intercept = −1.16, p <0.001), CN (intercept = −1.21, p <0.001), and MCI (intercept = −0.56, p =0.053), as well as for the OR comparing CN vs SCD (intercept = −0.27, p =0.037). No evidence of asymmetry was found for the OR comparing MCI vs SCD.

**b1. Leave-one-out influence diagnostics: proportion of APOE ε4 carriers in SCD (k = 49)**

Thresholds: |DFFITS| > 0.433; Cook’s D > 0.4549 (P(χ²) > 0.50); hat > 0.0612; |DFBETAS| > 1. Influential studies are highlighted in red.

| **Study** | **Hat** | **DFFITS** | **Cook’s D** | **DFBETAS** | **Pooled proportion without study** | **95% CI** | **Criteria exceeded** |
| --- | --- | --- | --- | --- | --- | --- | --- |
| Lautenschlager 2005 | 0.0202 | -0.115 | 0.0131 | -0.114 | 0.2855 | 0.2524–0.3209 | — |
| Kryscio 2014 | 0.0230 | 0.024 | 0.0006 | 0.019 | 0.2831 | 0.2500–0.3188 | — |
| Mandecka 2016 | 0.0201 | 0.053 | 0.0028 | 0.048 | 0.2826 | 0.2496–0.3181 | — |
| Wolfsgruber 2016 | 0.0202 | -0.138 | 0.0186 | -0.135 | 0.2858 | 0.2529–0.3212 | — |
| Fladby 2017 | 0.0218 | 0.128 | 0.0161 | 0.124 | 0.2813 | 0.2486–0.3164 | — |
| Reijs 2017 | 0.0211 | 0.200 | 0.0382 | 0.199 | 0.2801 | 0.2480–0.3146 | — |
| **Sierra-Rio 2017** | **0.0181** | **0.463** | **0.1743** | **0.487** | **0.2760** | **0.2471–0.3069** | **DFFITS** |
| Van Harten 2017 | 0.0222 | 0.162 | 0.0255 | 0.159 | 0.2807 | 0.2483–0.3156 | — |
| De Rojas 2018 | 0.0218 | -0.031 | 0.0010 | -0.035 | 0.2841 | 0.2509–0.3198 | — |
| Dubois 2018 | 0.0222 | -0.133 | 0.0174 | -0.131 | 0.2857 | 0.2527–0.3212 | — |
| Muller-Gerards 2019 | 0.0224 | 0.001 | 0.0000 | -0.003 | 0.2835 | 0.2503–0.3192 | — |
| Baldacci 2020 | 0.0222 | -0.131 | 0.0168 | -0.129 | 0.2857 | 0.2527–0.3212 | — |
| Bessi 2020 | 0.0174 | 0.057 | 0.0032 | 0.053 | 0.2825 | 0.2497–0.3179 | — |
| Hu 2020 | 0.0215 | -0.142 | 0.0198 | -0.140 | 0.2859 | 0.2529–0.3213 | — |
| Lee 2020 | 0.0217 | -0.047 | 0.0022 | -0.050 | 0.2843 | 0.2511–0.3200 | — |
| Mazzeo 2020 | 0.0213 | -0.004 | 0.0000 | -0.009 | 0.2836 | 0.2505–0.3193 | — |
| Stockmann 2020 | 0.0221 | 0.117 | 0.0136 | 0.113 | 0.2815 | 0.2488–0.3167 | — |
| Yoon 2020 | 0.0196 | -0.097 | 0.0093 | -0.097 | 0.2852 | 0.2521–0.3207 | — |
| Ayton 2021 | 0.0222 | 0.131 | 0.0168 | 0.127 | 0.2813 | 0.2486–0.3164 | — |
| Chen 2021 | 0.0199 | 0.043 | 0.0018 | 0.038 | 0.2828 | 0.2498–0.3183 | — |
| Hestad 2021 | 0.0229 | 0.158 | 0.0243 | 0.155 | 0.2808 | 0.2483–0.3157 | — |
| Janssen 2021 | 0.0234 | -0.001 | 0.0000 | -0.005 | 0.2836 | 0.2503–0.3193 | — |
| Hao 2022 | 0.0224 | -0.020 | 0.0004 | -0.024 | 0.2839 | 0.2507–0.3196 | — |
| Huang 2022 | 0.0207 | 0.017 | 0.0003 | 0.012 | 0.2832 | 0.2501–0.3188 | — |
| Moschini 2022 | 0.0184 | 0.033 | 0.0011 | 0.029 | 0.2829 | 0.2500–0.3184 | — |
| Tippett 2022 | 0.0193 | 0.172 | 0.0288 | 0.171 | 0.2805 | 0.2483–0.3152 | — |
| Zhou 2022 | 0.0205 | 0.062 | 0.0038 | 0.057 | 0.2825 | 0.2495–0.3179 | — |
| Zorkina 2022 | 0.0202 | -0.125 | 0.0155 | -0.124 | 0.2856 | 0.2526–0.3210 | — |
| Caillaud 2023 | 0.0179 | -0.104 | 0.0107 | -0.104 | 0.2853 | 0.2523–0.3207 | — |
| Cui 2023 | 0.0229 | -0.067 | 0.0045 | -0.069 | 0.2847 | 0.2515–0.3204 | — |
| Hong 2023 | 0.0198 | -0.151 | 0.0224 | -0.148 | 0.2860 | 0.2532–0.3213 | — |
| Lee 2023 | 0.0203 | 0.183 | 0.0324 | 0.182 | 0.2803 | 0.2481–0.3150 | — |
| Swinford 2023 | 0.0146 | 0.041 | 0.0017 | 0.038 | 0.2828 | 0.2500–0.3181 | — |
| Xu 2023 | 0.0229 | -0.319 | 0.0902 | -0.298 | 0.2884 | 0.2567–0.3224 | — |
| Baquero 2024 | 0.0198 | -0.276 | 0.0704 | -0.260 | 0.2878 | 0.2558–0.3221 | — |
| Blömeke 2024 | 0.0214 | 0.039 | 0.0015 | 0.034 | 0.2829 | 0.2498–0.3184 | — |
| Cammisuli 2024 | 0.0128 | -0.054 | 0.0029 | -0.056 | 0.2844 | 0.2516–0.3197 | — |
| Hsieh 2024 | 0.0143 | -0.129 | 0.0164 | -0.127 | 0.2857 | 0.2529–0.3208 | — |
| **Jurasova 2024** | **0.0122** | **0.452** | **0.1800** | **0.463** | **0.2763** | **0.2473–0.3073** | **DFFITS** |
| Kang 2024 | 0.0232 | -0.080 | 0.0065 | -0.082 | 0.2849 | 0.2517–0.3206 | — |
| Morrison 2024 | 0.0231 | -0.054 | 0.0029 | -0.056 | 0.2845 | 0.2512–0.3202 | — |
| Popescu 2024 | 0.0203 | 0.157 | 0.0241 | 0.155 | 0.2808 | 0.2484–0.3156 | — |
| Roh 2024 | 0.0152 | -0.160 | 0.0251 | -0.156 | 0.2861 | 0.2535–0.3212 | — |
| Yu 2024 | 0.0213 | 0.075 | 0.0056 | 0.070 | 0.2822 | 0.2493–0.3177 | — |
| Zhen Liu 2024 | 0.0213 | -0.165 | 0.0264 | -0.160 | 0.2862 | 0.2534–0.3215 | — |
| Alanko 2025 | 0.0215 | 0.050 | 0.0026 | 0.046 | 0.2827 | 0.2496–0.3182 | — |
| Giacomucci 2025 | 0.0228 | -0.005 | 0.0000 | -0.010 | 0.2836 | 0.2504–0.3194 | — |
| Marquez 2025 | 0.0235 | -0.093 | 0.0087 | -0.094 | 0.2851 | 0.2519–0.3208 | — |
| Zurron 2026 | 0.0201 | -0.130 | 0.0167 | -0.128 | 0.2857 | 0.2527–0.3211 | — |

**b2. Leave-one-out influence diagnostics: proportion of APOE ε4 carriers in CN (k = 18)**

Thresholds: |DFFITS| > 0.7276; Cook’s D > 0.4549 (P(χ²) > 0.50); hat > 0.1667; |DFBETAS| > 1. Influential studies are highlighted in red.

| **Study** | **Hat** | **DFFITS** | **Cook’s D** | **DFBETAS** | **Pooled proportion without study** | **95% CI** | **Criteria exceeded** |
| --- | --- | --- | --- | --- | --- | --- | --- |
| Muller-Gerards 2019 | 0.0781 | -0.189 | 0.0350 | -0.170 | 0.2240 | 0.1871–0.2659 | — |
| Hu 2020 | 0.0678 | -0.461 | 0.1731 | -0.439 | 0.2288 | 0.1941–0.2675 | — |
| Yoon 2020 | 0.0517 | -0.028 | 0.0008 | -0.023 | 0.2211 | 0.1848–0.2622 | — |
| Ayton 2021 | 0.0781 | 0.465 | 0.1733 | 0.428 | 0.2128 | 0.1796–0.2503 | — |
| Chen 2021 | 0.0408 | -0.212 | 0.0429 | -0.203 | 0.2245 | 0.1889–0.2646 | — |
| Hao 2022 | 0.0700 | -0.044 | 0.0019 | -0.036 | 0.2214 | 0.1844–0.2633 | — |
| Huang 2022 | 0.0625 | 0.053 | 0.0028 | 0.053 | 0.2196 | 0.1831–0.2609 | — |
| Zhou 2022 | 0.0465 | 0.062 | 0.0037 | 0.062 | 0.2194 | 0.1836–0.2601 | — |
| Zorkina 2022 | 0.0312 | -0.149 | 0.0216 | -0.144 | 0.2234 | 0.1879–0.2634 | — |
| Caillaud 2023 | 0.0335 | -0.040 | 0.0016 | -0.037 | 0.2213 | 0.1857–0.2617 | — |
| Cui 2023 | 0.0745 | -0.181 | 0.0322 | -0.164 | 0.2239 | 0.1870–0.2657 | — |
| Swinford 2023 | 0.0422 | 0.426 | 0.1626 | 0.395 | 0.2136 | 0.1810–0.2502 | — |
| Blömeke 2024 | 0.0398 | -0.093 | 0.0085 | -0.088 | 0.2223 | 0.1864–0.2629 | — |
| Cammisuli 2024 | 0.0211 | -0.025 | 0.0006 | -0.023 | 0.2211 | 0.1858–0.2608 | — |
| **Popescu 2024** | **0.0645** | **0.798** | **0.3954** | **0.727** | **0.2093** | **0.1810–0.2408** | **DFFITS** |
| Zhen Liu 2024 | 0.0602 | -0.067 | 0.0045 | -0.060 | 0.2218 | 0.1852–0.2633 | — |
| Marquez 2025 | 0.0803 | -0.070 | 0.0050 | -0.059 | 0.2218 | 0.1846–0.2642 | — |
| Zurron 2026 | 0.0571 | -0.055 | 0.0030 | -0.049 | 0.2216 | 0.1851–0.2630 | — |

**b3. Leave-one-out influence diagnostics: proportion of APOE ε4 carriers in MCI (k = 19)**

Thresholds: |DFFITS| > 0.7071; Cook’s D > 0.4549 (P(χ²) > 0.50); hat > 0.1579; |DFBETAS| > 1. Influential studies are highlighted in red.

| **Study** | **Hat** | **DFFITS** | **Cook’s D** | **DFBETAS** | **Pooled proportion without study** | **95% CI** | **Criteria exceeded** |
| --- | --- | --- | --- | --- | --- | --- | --- |
| Mandecka 2016 | 0.0526 | -0.174 | 0.0296 | -0.168 | 0.4155 | 0.3558–0.4778 | — |
| Reijs 2017 | 0.0614 | 0.080 | 0.0065 | 0.069 | 0.4081 | 0.3477–0.4714 | — |
| Sierra-Rio 2017 | 0.0543 | 0.325 | 0.0952 | 0.313 | 0.4009 | 0.3441–0.4605 | — |
| Van Harten 2017 | 0.0597 | 0.388 | 0.1286 | 0.375 | 0.3992 | 0.3433–0.4578 | — |
| Yoon 2020 | 0.0480 | -0.386 | 0.1322 | -0.356 | 0.4208 | 0.3641–0.4796 | — |
| Ayton 2021 | 0.0607 | 0.221 | 0.0466 | 0.205 | 0.4039 | 0.3450–0.4656 | — |
| Chen 2021 | 0.0480 | 0.002 | 0.0000 | -0.004 | 0.4104 | 0.3504–0.4731 | — |
| Hestad 2021 | 0.0627 | 0.191 | 0.0356 | 0.176 | 0.4048 | 0.3454–0.4671 | — |
| Hao 2022 | 0.0538 | -0.050 | 0.0024 | -0.053 | 0.4119 | 0.3516–0.4751 | — |
| Caillaud 2023 | 0.0402 | 0.005 | 0.0000 | 0.000 | 0.4103 | 0.3508–0.4725 | — |
| Cui 2023 | 0.0614 | -0.437 | 0.1589 | -0.401 | 0.4220 | 0.3655–0.4807 | — |
| Swinford 2023 | 0.0371 | 0.142 | 0.0197 | 0.135 | 0.4061 | 0.3477–0.4673 | — |
| Blömeke 2024 | 0.0539 | 0.083 | 0.0069 | 0.073 | 0.4079 | 0.3480–0.4708 | — |
| Cammisuli 2024 | 0.0321 | 0.338 | 0.1056 | 0.330 | 0.4006 | 0.3453–0.4586 | — |
| Roh 2024 | 0.0591 | -0.427 | 0.1541 | -0.392 | 0.4218 | 0.3652–0.4805 | — |
| Yu 2024 | 0.0582 | -0.019 | 0.0004 | -0.025 | 0.4110 | 0.3504–0.4745 | — |
| Zhen Liu 2024 | 0.0535 | -0.015 | 0.0002 | -0.021 | 0.4109 | 0.3506–0.4740 | — |
| Alanko 2025 | 0.0502 | 0.057 | 0.0032 | 0.048 | 0.4087 | 0.3488–0.4715 | — |
| Zurron 2026 | 0.0530 | -0.385 | 0.1308 | -0.356 | 0.4208 | 0.3638–0.4800 | — |

**b4. Leave-one-out influence diagnostics: OR CN vs SCD (k = 18)**

Thresholds: |DFFITS| > 0.7276; Cook’s D > 0.4549 (P(χ²) > 0.50); hat > 0.1667; |DFBETAS| > 1. Influential studies are highlighted in red.

| **Study** | **Hat** | **DFFITS** | **Cook’s D** | **DFBETAS** | **Pooled OR without study** | **95% CI** | **Criteria exceeded** |
| --- | --- | --- | --- | --- | --- | --- | --- |
| **Marquez 2025** | **0.2215** | **1.423** | **0.4317** | **0.887** | **0.7354** | **0.6465–0.8366** | **DFFITS; Hat** |
| Blömeke 2024 | 0.0230 | -0.157 | 0.0241 | -0.155 | 0.7877 | 0.6908–0.8982 | — |
| Cammisuli 2024 | 0.0071 | 0.021 | 0.0004 | 0.023 | 0.7784 | 0.6828–0.8874 | — |
| Popescu 2024 | 0.0427 | 0.186 | 0.0334 | 0.186 | 0.7697 | 0.6728–0.8805 | — |
| Swinford 2023 | 0.0136 | 0.136 | 0.0183 | 0.137 | 0.7725 | 0.6773–0.8810 | — |
| Zhen Liu 2024 | 0.0442 | 0.311 | 0.0923 | 0.293 | 0.7643 | 0.6695–0.8725 | — |
| Caillaud 2023 | 0.0146 | 0.067 | 0.0044 | 0.071 | 0.7759 | 0.6799–0.8853 | — |
| Cui 2023 | 0.1126 | -0.161 | 0.0257 | -0.124 | 0.7867 | 0.6816–0.9079 | — |
| Hao 2022 | 0.0777 | -0.090 | 0.0081 | -0.068 | 0.7834 | 0.6811–0.9009 | — |
| Huang 2022 | 0.0431 | -0.043 | 0.0019 | -0.031 | 0.7813 | 0.6822–0.8947 | — |
| Zhou 2022 | 0.0262 | -0.074 | 0.0053 | -0.067 | 0.7831 | 0.6856–0.8945 | — |
| Zorkina 2022 | 0.0157 | -0.002 | 0.0000 | 0.002 | 0.7795 | 0.6830–0.8895 | — |
| Ayton 2021 | 0.1039 | -0.057 | 0.0035 | -0.030 | 0.7813 | 0.6768–0.9020 | — |
| Chen 2021 | 0.0211 | -0.248 | 0.0597 | -0.254 | 0.7927 | 0.6968–0.9017 | — |
| Hu 2020 | 0.0589 | -0.159 | 0.0242 | -0.142 | 0.7873 | 0.6872–0.9020 | — |
| Yoon 2020 | 0.0277 | 0.118 | 0.0137 | 0.122 | 0.7731 | 0.6766–0.8834 | — |
| **Muller-Gerards 2019** | **0.1122** | **-0.829** | **0.3969** | **-1.018** | **0.8268** | **0.7383–0.9258** | **DFFITS; DFBETAS** |
| Zurron 2026 | 0.0341 | 0.189 | 0.0345 | 0.188 | 0.7697 | 0.6736–0.8795 | — |

**b5. Leave-one-out influence diagnostics: OR MCI vs SCD (k = 19)**

Thresholds: |DFFITS| > 0.7071; Cook’s D > 0.4549 (P(χ²) > 0.50); hat > 0.1579; |DFBETAS| > 1. Influential studies are highlighted in red.

| **Study** | **Hat** | **DFFITS** | **Cook’s D** | **DFBETAS** | **Pooled OR without study** | **95% CI** | **Criteria exceeded** |
| --- | --- | --- | --- | --- | --- | --- | --- |
| Alanko 2025 | 0.0495 | 0.062 | 0.0040 | 0.037 | 1.4723 | 1.2174–1.7806 | — |
| Blömeke 2024 | 0.0557 | 0.128 | 0.0166 | 0.103 | 1.4629 | 1.2104–1.7680 | — |
| Cammisuli 2024 | 0.0157 | 0.290 | 0.0805 | 0.311 | 1.4378 | 1.2108–1.7074 | — |
| Roh 2024 | 0.0333 | 0.081 | 0.0065 | 0.066 | 1.4684 | 1.2195–1.7681 | — |
| Swinford 2023 | 0.0200 | 0.080 | 0.0063 | 0.073 | 1.4676 | 1.2227–1.7616 | — |
| Yu 2024 | 0.0638 | -0.117 | 0.0135 | -0.137 | 1.4976 | 1.2347–1.8166 | — |
| Zhen Liu 2024 | 0.0543 | 0.573 | 0.2315 | 0.664 | 1.4014 | 1.1987–1.6383 | — |
| Caillaud 2023 | 0.0276 | 0.206 | 0.0406 | 0.208 | 1.4497 | 1.2119–1.7342 | — |
| Cui 2023 | 0.0888 | -0.385 | 0.1285 | -0.341 | 1.5258 | 1.2687–1.8350 | — |
| Hao 2022 | 0.0613 | 0.100 | 0.0105 | 0.070 | 1.4675 | 1.2114–1.7776 | — |
| Ayton 2021 | 0.0778 | 0.089 | 0.0089 | 0.050 | 1.4702 | 1.2089–1.7879 | — |
| Chen 2021 | 0.0401 | -0.001 | 0.0000 | -0.022 | 1.4807 | 1.2260–1.7884 | — |
| Hestad 2021 | 0.0938 | -0.047 | 0.0020 | -0.083 | 1.4901 | 1.2197–1.8204 | — |
| Yoon 2020 | 0.0391 | -0.137 | 0.0184 | -0.147 | 1.4985 | 1.2431–1.8063 | — |
| Reijs 2017 | 0.0692 | -0.381 | 0.1276 | -0.335 | 1.5238 | 1.2725–1.8249 | — |
| Sierra-Rio 2017 | 0.0406 | -0.529 | 0.2385 | -0.400 | 1.5276 | 1.2973–1.7988 | — |
| Van Harten 2017 | 0.0749 | 0.250 | 0.0591 | 0.227 | 1.4459 | 1.1987–1.7441 | — |
| Mandecka 2016 | 0.0470 | -0.234 | 0.0525 | -0.227 | 1.5096 | 1.2550–1.8158 | — |
| Zurron 2026 | 0.0476 | -0.053 | 0.0027 | -0.075 | 1.4884 | 1.2303–1.8006 | — |

SCD: subjective cognitive decline; CN: cognitively normal; MCI: mild cognitive impairment; OR: odds ratio; CI: confidence interval; DFFITS: difference in fits; DFBETAS: difference in regression coefficients; D: distance; hat: leverage value.

**c. Egger’s regression test for funnel plot asymmetry**

| **Analysis** | **k** | **Intercept** | **SE** | **Z** | **p** | **Significant (p<0.10)** |
| --- | --- | --- | --- | --- | --- | --- |
| SCD proportion | 49 | -1.16 | 0.19 | -6.20 | 0.001 | Yes |
| CN proportion | 18 | -1.21 | 0.21 | -5.70 | 0.001 | Yes |
| MCI proportion | 19 | -0.56 | 0.29 | -1.94 | 0.053 | Yes |
| OR CN vs SCD | 18 | -0.27 | 0.13 | -2.09 | 0.037 | Yes |
| OR MCI vs SCD | 19 | 0.06 | 0.24 | 0.25 | 0.802 | No |

**d. Duval and Tweedie’s trim-and-fill**

“Trim-and-fill” analyses imputed two studies in both the SCD and CN models, with negligible changes in the pooled estimates. No missing studies were imputed for the MCI proportion or the OR for CN vs SCD. For the OR comparing MCI vs SCD, one study was imputed on the right side, resulting in an adjusted OR of 1.44 compared with the original 1.48. In all cases, the adjusted estimates remained virtually unchanged and retained their statistical significance.

| **Analysis** | **k₀ imputed** | **Original estimate [95% CI]** | **Adjusted estimate [95% CI]** |
| --- | --- | --- | --- |
| SCD proportion | 4 | 0.284 [0.251; 0.318] | 0.268 [0.230; 0.309] |
| CN proportion | 3 | 0.221 [0.186; 0.259] | 0.231 [0.196; 0.269] |
| MCI proportion | 0 | 0.410 [0.353; 0.470] | 0.410 [0.353; 0.470] |
| OR CN vs SCD | 1 | 0.78 [0.68; 0.89] | 0.77 [0.68; 0.88] |
| OR MCI vs SCD | - | - | - |

Note: Trim-and-fill was applied only to models with significant Egger’s test (p < 0.10). k₀: number of imputed studies.

**Supplementary Figure S1. Forest plot of the pooled proportion of APOE ε4 carriers among individuals with MCI**

**Supplementary Figure S1. Forest plot of the pooled proportion of APOE ε4 carriers among CN individuals**
